# Pleiotropic Roles of a Ribosomal Protein in *Dictyostelium discoideum*

**DOI:** 10.1371/journal.pone.0030644

**Published:** 2012-02-17

**Authors:** Smita Amarnath, Trupti Kawli, Smita Mohanty, Narayanaswamy Srinivasan, Vidyanand Nanjundiah

**Affiliations:** 1 Department of Molecular Reproduction, Development and Genetics, Indian Institute of Science, Bangalore, India; 2 Molecular Biophysics Unit, Indian Institute of Science, Bangalore, India; University of Dundee, United Kingdom

## Abstract

The cell cycle phase at starvation influences post-starvation differentiation and morphogenesis in *Dictyostelium discoideum*. We found that when expressed in *Saccharomyces cerevisiae*, a *D. discoideum* cDNA that encodes the ribosomal protein S4 (DdS4) rescues mutations in the cell cycle genes *cdc24*, *cdc42* and *bem1*. The products of these genes affect morphogenesis in yeast via a coordinated moulding of the cytoskeleton during bud site selection. *D. discoideum* cells that over- or under-expressed DdS4 did not show detectable changes in protein synthesis but displayed similar developmental aberrations whose intensity was graded with the extent of over- or under-expression. This suggested that DdS4 might influence morphogenesis via a stoichiometric effect – specifically, by taking part in a multimeric complex similar to the one involving Cdc24p, Cdc42p and Bem1p in yeast. In support of the hypothesis, the *S. cerevisiae* proteins Cdc24p, Cdc42p and Bem1p as well as their *D. discoideum* cognates could be co-precipitated with antibodies to DdS4. Computational analysis and mutational studies explained these findings: a C-terminal domain of DdS4 is the functional equivalent of an SH3 domain in the yeast scaffold protein Bem1p that is central to constructing the bud site selection complex. Thus in addition to being part of the ribosome, DdS4 has a second function, also as part of a multi-protein complex. We speculate that the existence of the second role can act as a safeguard against perturbations to ribosome function caused by spontaneous variations in DdS4 levels.

## Introduction

The origin of heterogeneity within groups of cells that are (to begin with) identical in their genotype and phenotype is a fundamental issue in developmental biology. Mutational studies that explore the links between genes, proteins and phenotypes rarely go beyond the single gene-single protein-single trait framework. The social amoeba *Dictyostelium discoideum* (Dd) provides an experimentally tractable system that is both simple and sufficiently intricate to enable an exploration of multifunctional roles of proteins during development.

Under laboratory conditions, genetically identical amoebae that have been raised in a common environment come together and, via complex shape changes, construct a polarised motile structure, the slug, which is made up of two spatially patterned cell types [1]. The slug differentiates into a fruiting body consisting of viable spores and dead stalk cells [2]. Three sources of pre-aggregation bias can bear on post-aggregation cell fate: the nutritional status of a cell, the phase of the cell cycle and the level of cellular calcium [3], [4], [5]. Calcium levels vary in a cell cycle phase-dependent manner [6]. The present study was initiated with a view to exploring the cell cycle-calcium link further. The genetics of the cell cycle in the yeast *Saccharomyces cerevisiae* (Sc) has been studied intensively and we decided to take advantage of the fact that the sequences of some *S. cerevisiae* and *D. discoideum* genes and proteins are rather similar [7]. Cross-complementation can take place between them: for example the *Ddcdc2* gene of *D. discoideum* can rescue the *cdc28* mutant phenotype in *S. cerevisiae* and *DdyakA* can substitute for *yak1* in *S. cerevisiae* [8], [9]. The other way round, the *clu1* gene of *S. cerevisiae* complements the *cluA^−^* mutation in *D. discoideum* [10].

In yeast, the morphogenetic process of bud initiation is an essential event of the cell division cycle. Many inputs go into the process of bud site selection – in particular, for the symmetry-breaking steps that lead to bud initiation [11], [12], [13]. ScCdc24p, ScCdc42p and ScBem1p function in concert as part of a complex. ScBem1p acts as a scaffold protein and mobilises the concerted and location-specific activities of ScCdc42p (a GTPase) and ScCdc24p (a guanine-nucleotide exchange factor). We began by looking for a gene known to be important in regulating the yeast cell cycle that also had a ‘calcium link’. A search of the literature showed that the *cdc24* gene of yeast fulfilled both criteria [14]. The *cdc24-4* mutant shows a cell cycle arrest phenotype in that it is defective in bud formation at 37°C (but not at 30°C); and the post-Start phase cell cycle arrest that it exhibits is accompanied by a large influx of calcium [14]. With this information in hand we decided to find out whether both defects could be overcome by transforming *cdc24-4* yeast cells with DNA from *D. discoideum*. We first describe how a *D. discoideum* gene capable of substituting for *cdc24* and other cell cycle mutants in *S. cerevisiae* was identified and characterised. An account of the unexpected consequences of over- or under-expressing the gene in *D. discoideum* follows next. The outcome led to a hypothesis regarding the manner in which the relevant gene products might function. Experiments were carried out to test the hypothesis, initially in *S. cerevisiae* and then in *D. discoideum*.

## Results

### cDNA encoding the D. discoideum S4 gene suppresses specific cell cycle mutations in S. cerevisiae

**Figure 1:**
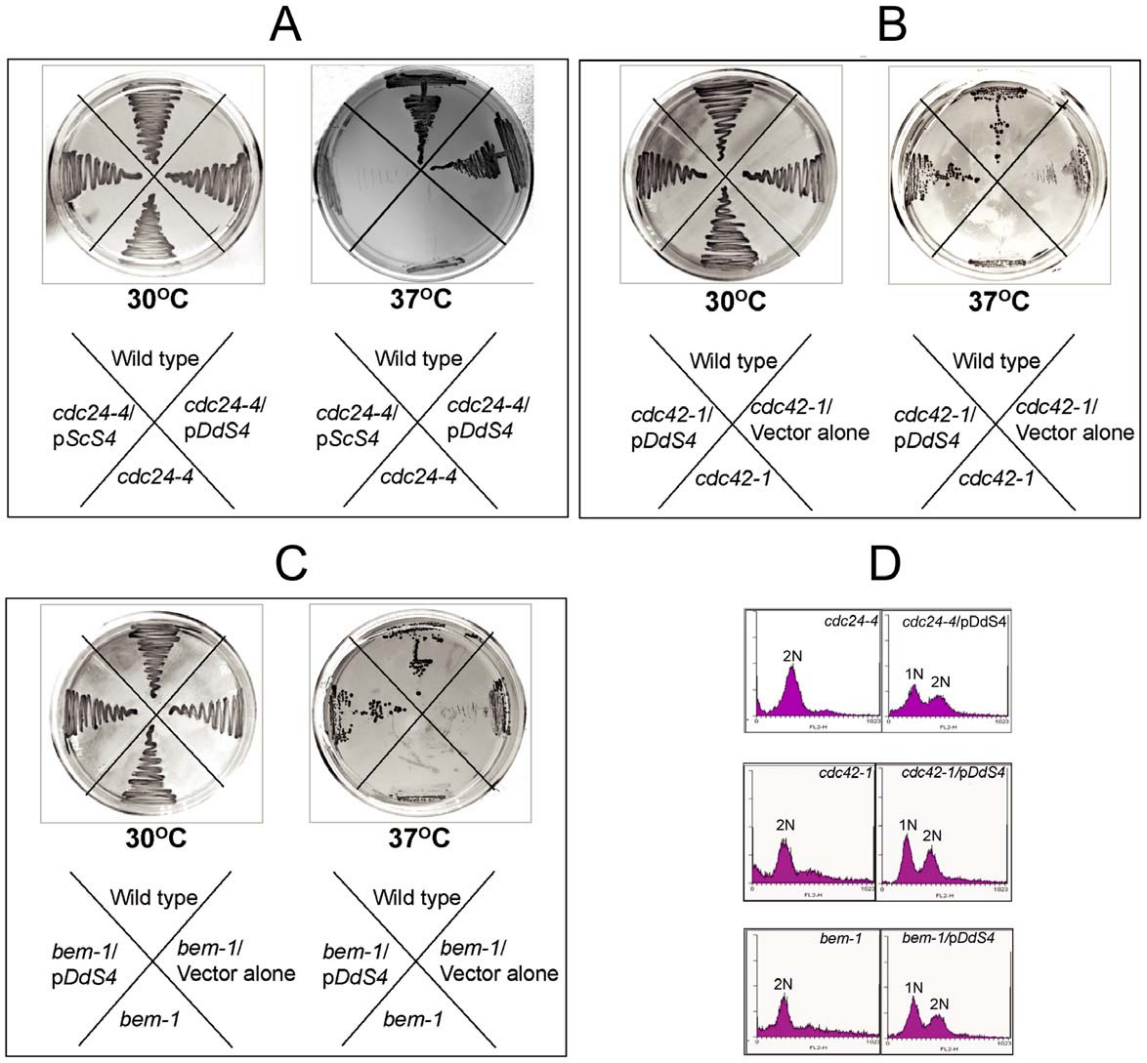
Rescue of temperature sensitive cell cycle mutants with DdS4. Growth of *S. cerevisiae* strains *cdc24-4*, (**B**) *cdc42-1*, (**C**) *bem1* transformed with control vector or *DdS4* or *ScS4* at 30°C and 37°C. (**A**) shows that *ScS4* is unable to rescue the cell cycle defect in *cdc24-4* (**A**). (**D**) Cell cycle phases monitored by FACS on propidium iodide-stained cells. Complemented strains show 2 peaks corresponding to 1 N and 2 N DNA content as compared to the mutants which show just the 2 N peak (indicative of cell cycle arrest).

We looked for complementation in an attempt to find functional equivalents of cell cycle genes in *D. discoideum*. Multiple *S. cerevisiae* mutants with defects in cell cycle progression were transformed with a vegetative phase cDNA library of *D. discoideum* (a kind gift from Dr. Catherine Pears). The *D. discoideum* cDNA library was constructed to function under the yeast alcohol dehydrogenase promoter. To begin with we used temperature-sensitive cell cycle mutants in *cdc24*, *cdc42* and *bem1*. Since the mutants were temperature sensitive for growth at 37°C and uracil auxotrophs, the transformants were screened at 37°C on a uracil-deficient plate [15]. Of the several independent transformants that were obtained, one was followed up for further study since it rescued the cell cycle defect in the *cdc24-4*, *cdc42-1* and *bem1* mutants (Figure 1). The 870 bp cDNA that rescued *cdc24*-2, *cdc42*-1 and *bem1* was found to encode for the *D. discoideum* ribosomal protein DdS4. The rescuing DNA constituted a full-length gene with no introns and was identical to the *S4* sequence reported in the literature [16]. A messenger RNA encoding *D. discoideum S4* was reported to be expressed selectively in prestalk cells [17]. However, that mRNA encodes a different ribosomal protein, S2; it does not encode S4 [18]. Another *S4* mRNA, which is said to be specifically expressed during the growth-to-differentiation transition in *D. discoideum*, is transcribed from the mitochondrial genome, and its sequence does not overlap to any significant extent with that of *DdS4* [19]. We examined whether the *S. cerevisiae* S4 (ScS4) was also able to rescue the yeast mutants mentioned above. Unlike *DdS4*, *ScS4* failed to complement any of the three (Figure 1A). This suggested to us that the observed rescue was specific to *DdS4*.

### D. discoideum S4 specifically suppresses cdc24-4, cdc42-1 and bem1 in S. cerevisiae

S4 is a component of the small subunit of the ribosome. It is present at a position where it can affect codon-anticodon interactions during translation and, thereby, translational fidelity. Mutations in S4 have been reported to result in non-specific increase in errors (missense, nonsense and frameshift) during translation in both *S. cerevisiae* and *Escherichia coli* [20], [21]. Since the phenotypes of *cdc24-4* and *cdc42-1* and *bem1* (disruptant) were all rescued by *DdS4* expression, we wondered whether DdS4 was inducing nonspecific errors during translation in *S. cerevisiae*. We addressed this concern by testing the effect of *DdS4* expression on the phenotypes of eight independent mutations comprising both missense and nonsense categories and located on different linkage groups in *S. cerevisiae*. None of the eight mutations were suppressed by *DdS4* (Table 1), suggesting that the rescue of the three cell cycle mutants we observed was a specific effect. These results pointed to an as yet unknown function for *DdS4*.

**Table 1:** DdS4 does not affect translational fidelity.

Marker	Nature	Error rate/cell
*his3-11*	nonsense	<10^−4^
*trp1-1*	?	<1.5×10^−5^
*lys2*	missense	<10^−4^
*leu2-3*	nonsense	<10^−4^
*cdc7-1*	missense	<10^−4^
*cdc31-1*	missense	<10^−4^
*cdc36-16*	missense	<10^−4^
*esp1*	missense	<10^−4^
*cdc24-4*	missense	1

Rescue of *S. cerevisiae cdc24-4* by *D. discoideum S4* is not due to non-specific translational error. Eight different *S. cerevisiae* strains (kindly made available by Dr. U. Surana) harbouring the mutant alleles shown were transformed with the λADH: *DdS4* plasmid. The transformants were analysed for reversion (10^4^ transformants for each allele).

### DdS4 can form a complex with proteins that regulate bud formation in S. cerevisiae

DdS4 had been picked up on the basis of its ability to rescue the *S. cerevisiae* cell cycle mutants *cdc24-4*, *cdc42-1* and *bem1*, which influence cytoskeletal polarisation and depolarisation at specific phases of the cell cycle (Figure 1). Polarity establishment requires that the small GTPase Cdc42p, its exchange factor Cdc24p, and a scaffold protein, Bem1p function co-ordinately as part of a complex. Subsequently, Cdc42p recruits a number of effectors, among them a kinase, Cla4p, which phosphorylates Cdc24p and renders it inactive. It is believed that ScCdc24p functions via a complex formed by ScBem1p, ScCla4p, ScCdc42p, and ScCdc24p [22]. Given the ability of *DdS4* to rescue the *cdc24-4*, *cdc42-1* and *bem1* cell cycle mutants, we wondered whether DdS4 could substitute for one or the other member of the complex formed by ScCdc24p, ScCdc42p and ScBem1p.

**Figure 2:**
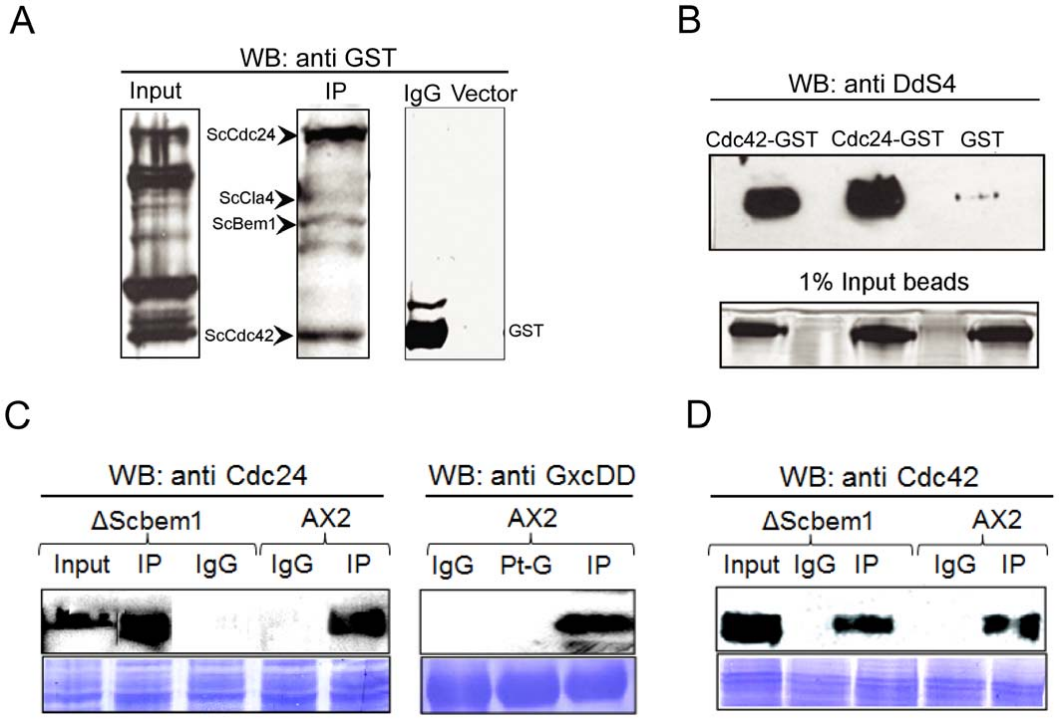
Western blots (WB) showing co-immunoprecipitation using DdS4 antibody; lower panels in C and D show Coomassie-stained proteins. (**A**) Cell lysates from cells over-expressing Cdc42p, Cdc24p, Bem1p and Cla4p as GST-tagged proteins were mixed in equal volumes. Subsequently upon specific interaction with DdS4 antibody the proteins were resolved on 12% SDS PAGE and probed with anti-GST antibodies. The proteins resolved on the gel were identified on the basis of their molecular weights. Specific interactions between the GST-tagged proteins and DdS4 are observed in the IP lane. Controls: IgG-pull down using normal rabbit IgG or vector-lysates from cells over-expressing the GST-tagged proteins and an empty vector, show no interactions. (**B**) Individual cell lysates from *E. coli* BL21 over-expressing Cdc42p and Cdc24p as GST-tagged proteins respectively were allowed to interact with GST (Glutathione S-transferase) beads subsequently the beads were incubated with a cell lysate over-expressing DdS4. The GST beads were run on 12% SDS PAGE and probed with anti-DdS4 antibody. The input panel shows that equal amount of beads were loaded. (**C**) and (**D**) Specific interactions studied using antibodies raised against DdS4 and commercial anti-CDC24 or anti-CDC42 antibodies. (**C**) Cell lysates from *S. cerevisiae bem1* or control AX2 *D. discoideum* cells were probed with anti-Cdc24 or anti-GxcDD antibody. IgG lane is a control with normal rabbit IgG. IP lane shows specific interaction when anti-DdS4 antibody is used for pull-down. Pt-G lane is the pull down using protein G beads alone. (**D**) Cell lysates from *bem1* or control AX2 cells probed with anti-Cdc42. The lanes are labeled as described above. Coomassie stained of the nitrocellulose blot shows equal loading.

We tested this hypothesis by using polyclonal anti-DdS4 antibody on whole cell lysates from *S. cerevisiae* cells that expressed DdS4 along with GST-tagged ScCdc24p, ScCdc42p, ScBem1p and ScCla4p proteins. Anti-DdS4 antibody was able to immunoprecipitate the yeast proteins ScCdc24p, ScCdc42p, ScBem1p and ScCla4p (Figure 2A & Figure S2B, I, II, III). In the case of ScCdc42p and ScCdc24p, this was based on DdS4 interacting separately with each, as shown by GST-pull down assays using either GST-tagged Cdc42p or Cdc24p (Figure 2B). Endogenous ScCdc42p and ScCdc24p in yeast lysates could also be immunoprecipitated by antibodies against DdS4 (Figure 2C & 2D). Anti-DdS4 antibodies were used to pull down proteins from *D. discoideum* cell lysates; the immunoprecipitate could be recognised by anti-human Cdc42 and anti-human Cdc24 antibodies (Figure 2C & 2D). Finally after immunoprecipitation of *D. discoideum* cell lysates with anti-DdS4 antibody we could detect the presence of the RacGEF GxcDD (Figure 2C); DdRacGEF proteins are the putative equivalents of ScCdc24p [23]. We went on to delineate the features of DdS4 that enabled it to specifically interact with the three cell cycle proteins in *S. cerevisiae* and *D. discoideum*.

### A shared SH3 domain between ScBem1p and DdS4 has functional implications

**Figure 3:**
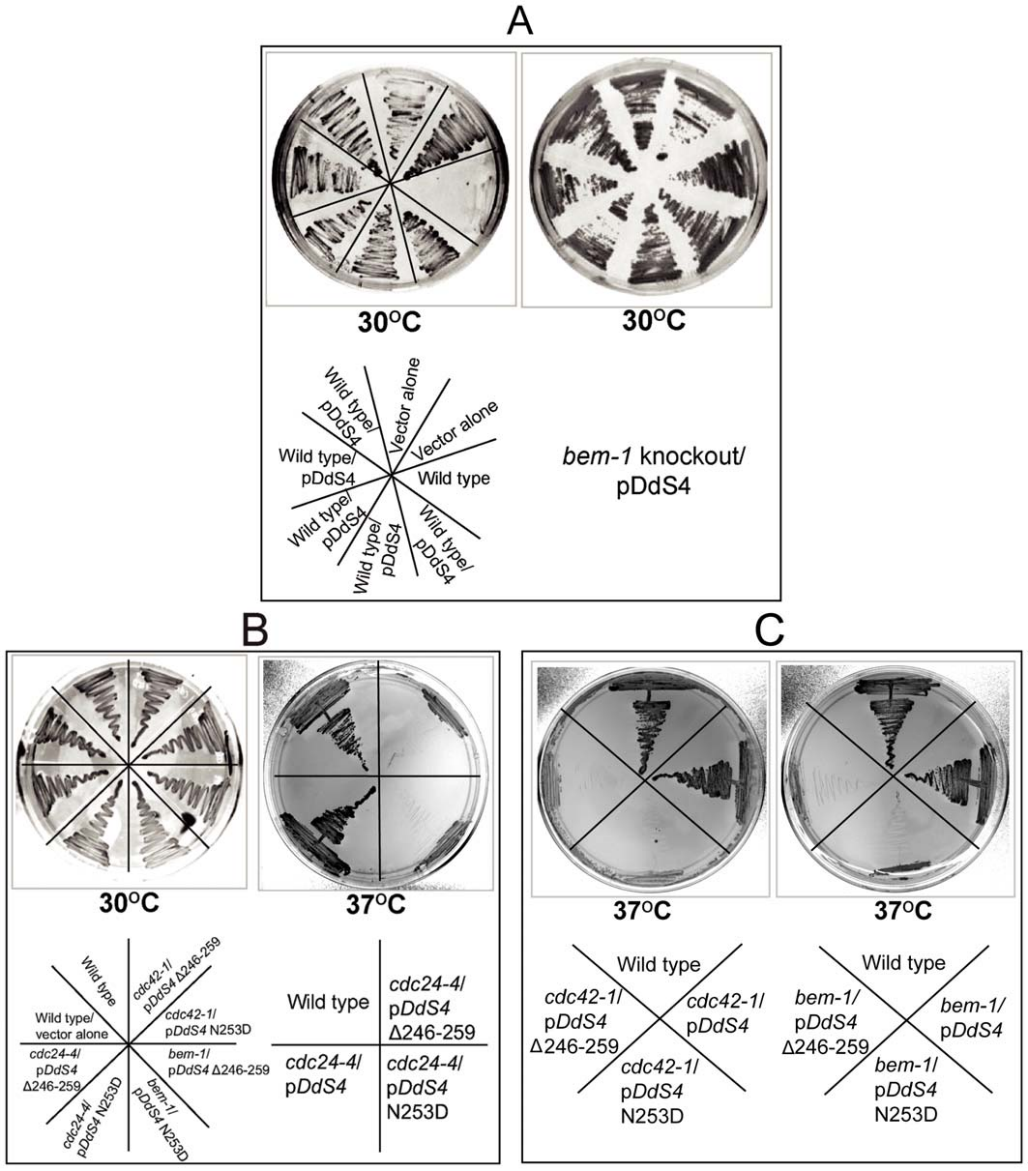
*bem1* knockout is rescued by DdS4. (**A**) The wild type control *S. cerevisiae* strain is unable to grow on a Ura^−^ dextrose plate whereas it can grow after transformation with vector alone or p*DdS4* containing plasmids on selective plates (left). Viable *bem1* knockout cells were obtained on Leu^−^ Ura^−^ dextrose plate only in the presence of pDdS4 plasmid (shown on right) or when *Scbem1* was provided on the plasmid (not shown). (**B**) and (**C**) The temperature sensitive cell cycle mutants *cdc24-4*, *cdc42-1* and *bem-1* are rescued by the full length DdS4. Expression of DdS4 harbouring a deletion of the C-terminal (246–259) or a point mutation (N253D) abolished the rescue.

In principle, DdS4 could participate in the yeast bud site selection complex by acting like a scaffold protein – for example, like ScBem1p, whose over-expression rescues the *Sccdc24* and *Sccdc42* mutant phenotypes [22], [24]. Since the *bem1* knockout in *S. cerevisiae* is lethal (Dr. D. Johnson, personal communication) [25], the following strategy was devised to test the possibility. A haploid *S. cerevisiae* strain harbouring a plasmid encoding either Sc*bem1* or *DdS4* was used to generate a knockout of *bem1* at the chromosomal locus. The reasoning was that if *DdS4* is able to substitute for *bem1* it would rescue the lethality due to absence of *bem1*. The expression of *DdS4* was indeed able to restore growth ability to the otherwise lethal *bem1* ^−/−^ strain {(ΔSc*bem1*) as, expectedly, was Sc*bem1* (data not shown)} (Figure 3A). Anti-DdS4 antibody was able to pull down endogenous Cdc42p and Cdc24p in *S. cerevisiae* cells lacking Bem1p (Figure 2C).

The genetic complementation and pull-down studies with yeast led us to ask if there could be a structural basis for the functional similarity between DdS4 and ScBem1p. Using hidden Markov models of protein domain families we carried out an analysis of the amino acid sequence of ScBem1p and DdS4 multi-domain proteins (Figure S1A) [26]. The C-terminal region of DdS4 comprising of the KOW motif is predicted to lie within the b.34.5 superfamily which corresponds to the all β-SH3-like translation protein domain folds (Figure S1A). We further analysed the C-terminal region of DdS4 to explore the extent to which it can mimic the role of SH3 domains in ScBem1p. Secondary structure prediction for the C-terminal region of DdS4 (150–267) showed predominantly extended β-sheets (Figure S1B). Residues 246–260 were predicted to be helical. The structural similarity of the predicted C-terminal portion of DdS4 to an SH3 domain was not obvious at the amino acid sequence level. The overall topology of the secondary structure was conserved between the domains of these two proteins based on fold recognition and modelling of the C-terminal region of DdS4 (Figure S1C). When compared with the SH3 domains from ScBem1p, a model of the DdS4 C-terminus resembled the second SH3 domain of ScBem1p more than it does the first (28.7% and 5.2% similarity respectively as obtained using Superpose [27]; the two SH3 domains in ScBem1p are considerably divergent in sequence). Multiple sequence alignment of close homologues of ScBem1p showed that the second SH3 domain and the following C-terminal region were well conserved. No other *D. discoideum* protein was identified that resembled ScBem1p in sequence, though the search did pick up other SH3 domain-containing proteins that resembled ScBem1p weakly (data not shown).

**Figure 4:**
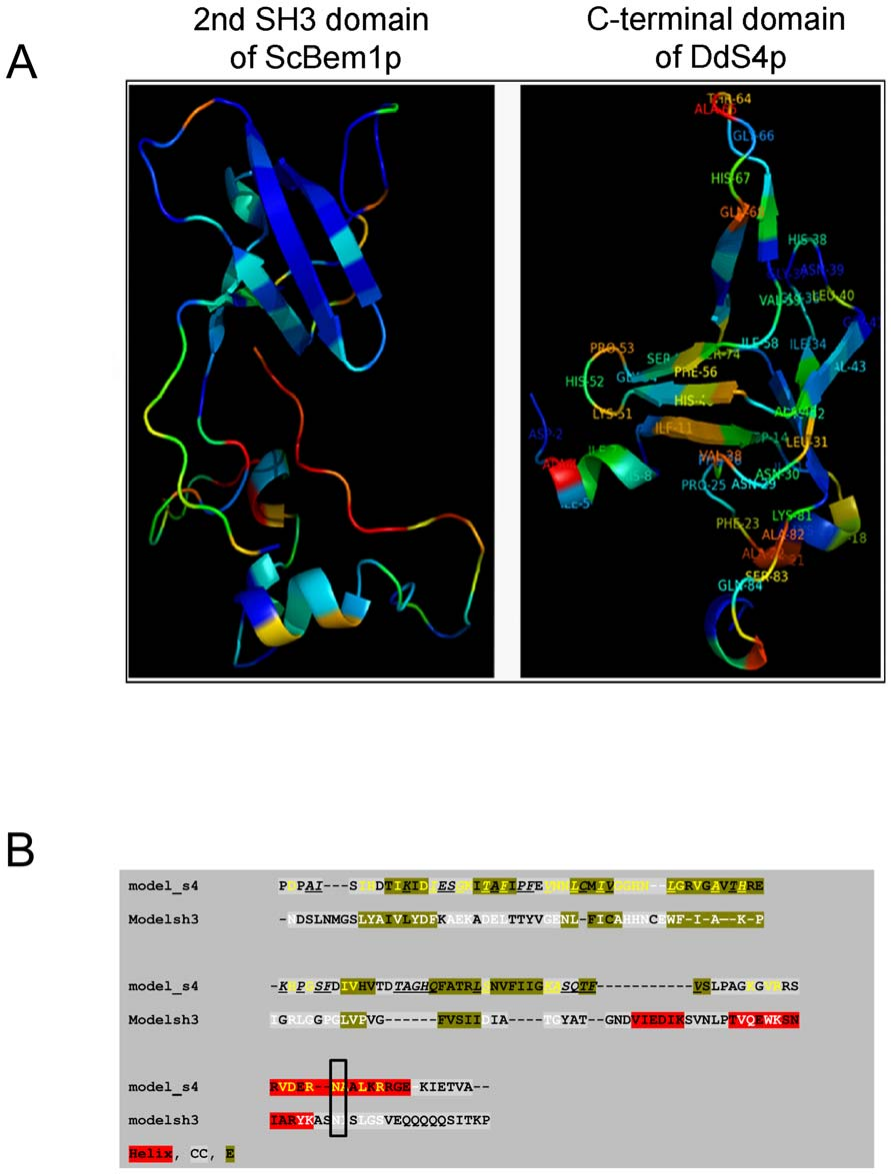
Structural model of the second SH3 domain of *S. cerevisiae* Bem1 and the C-terminal region of *D. discoideum* S4. (**A**) The structures have been color-coded based on the extent of conservation of residues amongst their homologs. Blue depicts the most conserved region while red represents the least conserved regions (annotation obtained from ConSurf) [76]. Residues conserved amongst Bem1 homologs (residues lying in Blue region) are labeled on structurally equivalent regions on the S4 C-terminal modeled structure. Note that not all the labeled residues are conserved amongst the S4 homologs (green, blue color). Many of the labeled residues lie in variable regions (orange, yellow, red colors). (**B**) Sequence alignment between the C-terminal region of Dds4 and the second SH3 domain of ScBem1 for which above modelled structures were generated. The secondary structural elements predicted for both the sequences are highlighted in red (helix), green (beta sheet/strand) and grey (coil) colours respectively. Residue positions in the alignment which are identical or conservatively substituted are highlighted in yellow and white for DdS4 and ScBem1 respectively. The black outlined box highlights the conserved Asn in the C-terminus which has been implicated in playing an important role in the functioning of the proteins.

Figure 4 shows the predicted model structure of the second SH3 domain of ScBem1p and C-terminus of DdS4. All the residues conserved in Bem1p are labelled on the DdS4 model. Region 178–235 of DdS4 aligns with both the SH3 domains and defines the structural core of the SH3-like region in DdS4. To test if the predicted structural core SH3-like region of DdS4 was functionally important we carried out a deletion analysis. If the DdS4 structural core SH3 domain with similarities to Bem1p was functionally relevant, its deletion should abolish the observed ability of DdS4 to rescue the cell cycle mutants. Deletion of amino acids 178–235 at the C terminal end of DdS4 failed to rescue the temperature sensitive phenotype of *cdc24-4*, *cdc42-1* and *bem1* mutants (Figure 3B & 3C).

Asparagine 253 of Bem1 is important in mediating interaction with Cdc42p; the N253D mutation abrogates interaction with Cdc42p [28]. Structure-based alignment with the SH3 domain fold region of DdS4 showed that this residue is equivalent to a conserved asparagine of DdS4 (Figure 4A & 4B). Remarkably when a similar mutation is introduced in DdS4 it loses its ability to rescue the *cdc24-4*, *cdc42-1* and *bem1* mutant phenotype (Figure 3B & 3C), indicating a functional role for this amino acid residue which is conserved in all higher eukaryotes starting from *D. discoideum* but is absent in *S. cerevisiae*. We next examined the possible roles of DdS4 in *D. discoideum* development

### DdS4 expression and localization in D. discoideum

**Figure 5:**
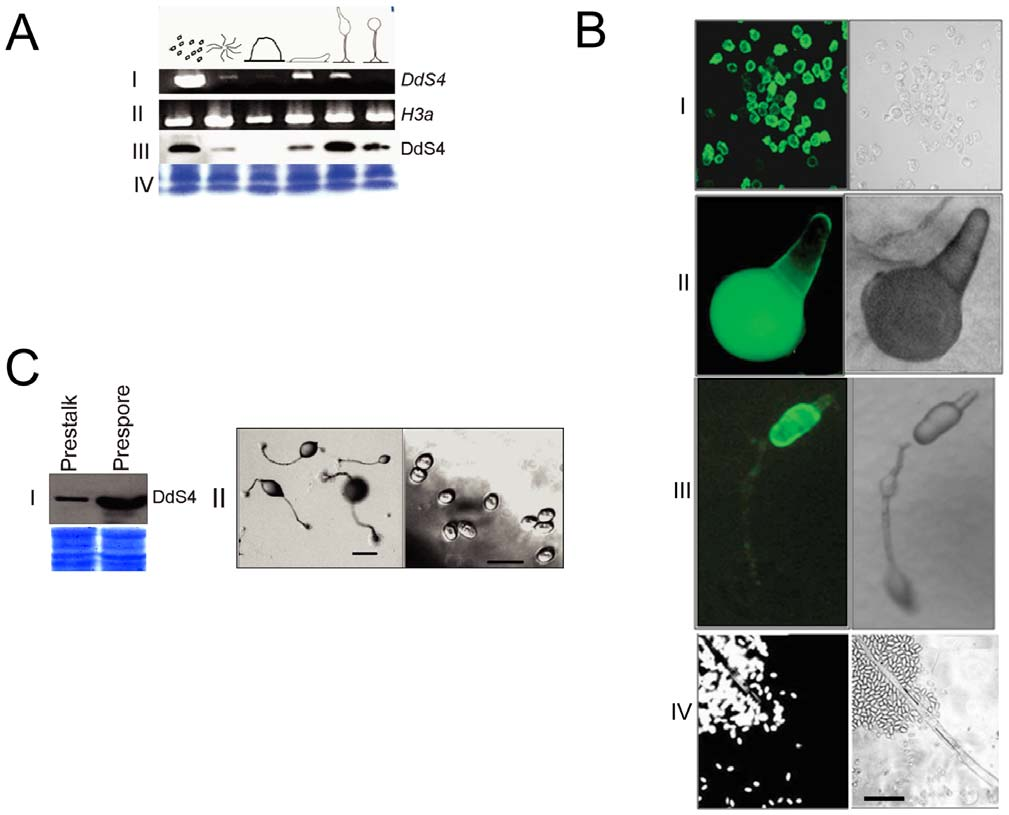
Expression profiles of DdS4 during *D. discoideum* development. (**A**) Temporal expression. **I.** Transcript levels of *DdS4* mRNA as visualized using RT-PCR. **II.** *H3a* mRNA levels used as loading control. **III.** Western blot using DdS4 specific antiserum. **IV** Coomassie stained gel shows the amount of protein loaded in each lane. Samples were obtained at approximately 0, 8, 14, 18, 20 and 24 hours of development and the corresponding developmental stages are shown. (**B**) Spatial expression. GFP fluorescence is indicative of the expression from a putative *DdS4* promoter. **I** Amoebae **II** Tipped mound **III** Late culminant **IV** Fruiting body and spores. Scale bar: 10 µm. (**C**) Cell-type localisation of DdS4 protein. **I** Equal amounts of protein obtained from the slug tissue corresponding to prestalk and prespore cells were run on 12% SDS PAGE and probed with anti-DdS4 antibody. Coomassie stained blots show equal loading of the protein. **II** Cell type-specific removal of DdS4 using psp promoter to analyse the importance of the predominant prespore localisation of DdS4. The left panel shows normal looking fruiting bodies however the spores formed are smaller, fewer, with low spore viability (right panel). Refer to Table 2.

The *DdS4* gene, which was isolated from a vegetative phase *D. discoideum* (AX2) cDNA library, showed a high level of expression during the growth phase (Figure 5A). *DdS4* genomic DNA 1 Kb upstream of the start codon was cloned in-frame to a GFP reporter and used to transform cells (*DdS4prm:GFP*). Growth phase transformants were strongly fluorescent (Figure 5B I). The PSORT software program which predicts subcellular protein localization [29] assigned a cytoplasmic localisation (p = 76%) to the DdS4 protein. Subcellular fractionation revealed that DdS4 was also associated with the TritonX-100 soluble and insoluble cytoskeletal fraction (Figure S2C).

Next we looked at the expression profile of *DdS4* during *D. discoideum* development. RT-PCR analysis showed that the expression of *DdS4* declined during post-starvation development and increased again later in the slug (Figure 5A). *DdS4prm:GFP* cells showed weak GFP expression during aggregation and the spatial pattern became restricted to the posterior prespore region at the tipped mound stage and beyond; fluorescent spores (but not stalk cells) were observed subsequently (Figure 5B II, III, IV). *In situ* hybridization confirmed this prespore and spore specific expression of *DdS4* (Figure S2A). The developmental profile of the DdS4 protein mirrored that of the transcript (Figure 5A). Western blot analysis using DdS4 specific antibody on disaggregated cells from slugs confirmed the predominantly prespore localization of DdS4 (Figure 5C I & Figure S2B - I & II; prespore-specific down-regulation of DdS4 resulted in fruiting bodies with fewer and smaller than normal spores; Figure 5C II and Table 2).

**Table 2:** The effect of DdS4 perturbation on spore formation and viability.

Strain	Spore forming efficiency (%)	Spore viability (%)
AX2	76.2±4.6	93.5±3.69
AX2:A15S4^+^_m_	7.8±1.5	5.3±0.75
AX2:A15S4^−^_m_	9.2±0.5	3.6±0.2
AX2:pspS4^−^	24±2.5	23±2

Spore forming efficiency and viability of AX2, AX2:A15S4^+^ and AX2:A15S4^−^. (Estimated on the basis of counting about 5000 spores per experiment for each strain. Mean ± S.D., N = 4).

### Similar developmental aberrations are seen irrespective of whether DdS4 is up- or down-regulated under a constitutive promoter

In order to understand the role of DdS4 in *D. discoideum* development, we attempted to knock out the S4 gene by homologous recombination. Numerous attempts to do so failed. We reasoned that a total lack of DdS4 function could compromise ribosomal function sufficiently to affect cellular viability. To circumvent this problem, we tried to modify the level of *DdS4* expression without eliminating it entirely.

**Figure 6:**
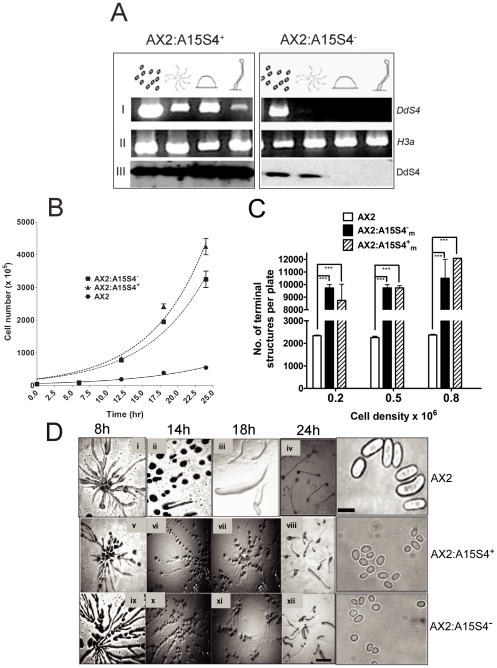
Phenotypes of DdS4 over-and under-expressors. (**A**) **I** and **II** Semi-quantitative RT-PCR using *DdS4* specific primers. **III** DdS4 protein expression profiles during development in DdS4 over-expressor (AX2:A15S4^+^) and DdS4 under-expressor (AX2:A15S4^−^). (**B**) Growth curves of AX2 and AX2:A15 transformants. Counts were taken of cells growing with *Klebsiella aerogenes* every 3 hours. Points indicate means of 3 independent counts ± S.D. (**C**) Aggregate sizes for AX2 and AX2:A15 transformants. All terminal structures (fruiting bodies in case of AX2 and fingers in case of AX2:A15 transformants) were counted. (Estimated on the basis of about 200 terminal structures per plate; Mean ± S.D., N = 4). The p values were calculated using one-way ANOVA and were <0.05. (**D**) Developmental phenotypes of *D. discoideum* AX2 (i–iv), AX2:A15S4^+^_m_ (intermediate over-expressor of DdS4; v–viii); and AX2:A15S4^−^_m_ (intermediate under-expressor of DdS4; ix–xii). Common scale bar for i–xii, 100 µm. The images in the last column are of spores formed by the wild type, AX2:A15S4^+^_m_ and AX2:A15S4^−^_m_ cells (scale bar, 10 µm).

Towards this end *DdS4* cDNA was cloned in antisense orientation under the *D. discoideum* actin15 promoter. Since DdS4 was expressed during the vegetative as well as the developmental phase, we choose the actin 15 promoter which is known to be expressed during both these stages [30]. Relative to controls (i.e. empty vector-containing transformants), the anti-sense construct (AX2:A15S4^−^) caused a lowering in both transcript and protein levels (Figure 6A, for control see Figure 5A). The AX2:A15S4^−^ cells divided faster than the controls and were smaller (Figure 6B and Table 3 & Table 4). They aggregated over a similar time-course (8–10 hours) as controls; however, the aggregation streams fragmented into small aggregates that proceeded to form slugs and, eventually, erect undifferentiated finger -like structures that occasionally had a tiny spore mass at the top (Figure 6C & 6D and Table 5). The spores were spherical, not elliptical as normally, and of lowered viability (Figure 6D and Table 2). Next we sought to see the effects of *DdS4* overexpression. We cloned the *DdS4* cDNA in the sense orientation, again under the Actin 15 promoter, and confirmed that ‘sense’ transformants (AX2:A15S4^+^) had increased levels of the *DdS4* transcript and DdS4 protein at all developmental stages (Figure 6A, for control see Figure 5A). To our surprise, the *DdS4* over-expressors had phenotypes strikingly similar to the antisense driven under-expressors (Figure 6B–D and Tables 2, 3, 4, 5).

**Table 3:** The effect of DdS4 perturbation on doubling time.

Strain	Doubling time (hrs.)
AX2	5.5
AX2:A15S4^+^_m_	3.9
AX2:A15S4^−^_m_	4.3

The mean doubling time for each of the strains was estimated from the exponential portion of the growth curves in Fig 2B (N = 3).

**Table 4:** The effect of DdS4 perturbation on cell size.

Strain	Cell size (arbitrary units)
AX2	0.194±0.01
AX2:A15S4^+^_m_	0.102±0.02
AX2:A15S4^−^_m_	0.105±0.03

Comparison of diameters of growth phase amoebae of AX2, AX2:A15S4^+^ and AX2:A15S4^−^ made by taking images at 10× magnification. An average of the cell lengths was measured transversely and longitudinally. 0.1 arbitrary unit equals 10 microns. Mean ± S.D. N = 3.

**Table 5:** The effect of DdS4 perturbation on aggregate size.

Initial cell density	No. of aggregates		
	AX2	AX2:A15S4^+^_m_	AX2:A15S4^−^_m_
2×10^5^/cm^2^	2340±43	9731±383	8740±1820
5×10^5^/cm^2^	2250±116	9739±374	9737±252
8×10^5^/cm^2^	2272±38	10500±2126	12082±26

Number of aggregates formed/20 cm^2^ at the indicated cell densities by AX2, AX2:A15S4^+^ and AX2:A15S4^−^. Mean ± S.D. [Based on ∼ 300 fruiting bodies (for AX2) and terminal structures (AX2:A15S4^+^ and AX2:A15S4^−^) counted in replicate plates; N = 4.]

### Ribosomal function remains largely unperturbed when DdS4 is up- or down-regulated

**Figure 7:**
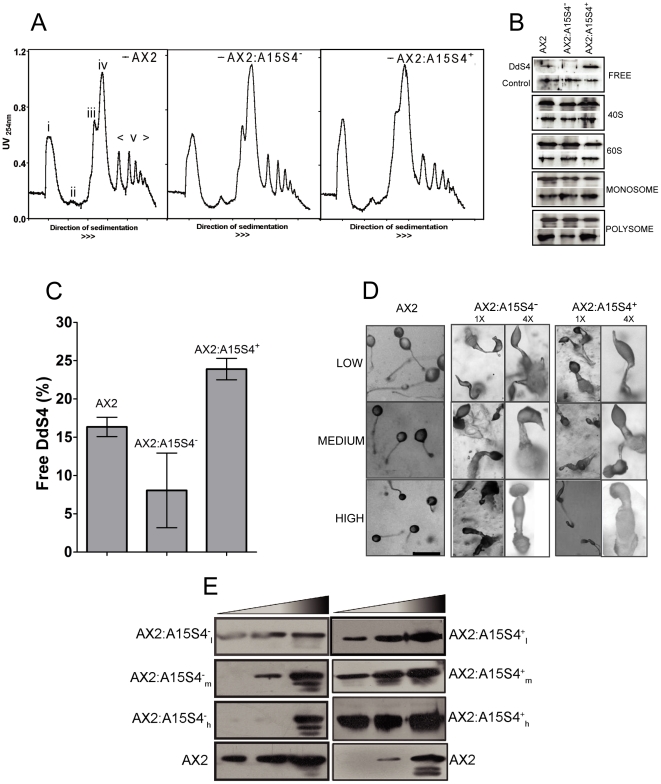
AX2:A15 transformants show normal ribosomal function and display phenotypic variability. (**A**) Polysome profiles of AX2, AX2:A15S4^+^_m_ (intermediate over-expressor of DdS4) and AX2:A15S4^−^_m_ (intermediate under-expressor of DdS4) cells. **i** Free protein **ii** 40S subunit **iii** 60S subunit **iv** Monosomes **v** Polysomes. The analysis was repeated twice with independently isolated polysomes for each strain. (**B**) Fractions corresponding to different peaks in the polysome profile were collected and subjected to TCA precipitation. Equal amounts of protein from each fraction were run on a 12% SDS PAGE. Western blot analysis was carried out using anti-DdS4 antibody. In all panels the protein band in the lane below is the normalising control. (**C**) Quantitation of DdS4 in terms of net intensity of the free fraction has been represented in histograms. The band intensities of independent exposures of Western blots were measured using Quantity One analysis software from BioRad. p values were calculated using one-way ANOVA and were <0.05. (**D**) Terminal structures at low and high magnification (‘low’, ‘medium’ and ‘high’ over-expressors on the right and under-expressors on the left). Scale bar: 100 µm. For better clarity magnified images (denoted as 4×) are shown in the panels on the right. (**E**) Western blots showing expression levels of DdS4 in AX2, AX2:A15S4^+^ and AX2:A15S4^−^ cells. The total protein taken for over-expressors is lesser than that taken for under-expressors so as to prevent saturation of the band intensities.

Our analysis in *S. cerevisiae* indicated that the DdS4-induced rescue of mutant phenotypes was unlikely to be due to aberrations in ribosomal function. We wanted to verify that this was true also in the case of *D. discoideum*. We used polyribosomal profiling as a means of measuring overall translational efficiency [31]. Sucrose density gradient centrifugation of stalled ribosome complexes (harbouring peptidyl tRNAs) resolves them into fractions corresponding to monosomes (70S ribosomes) and polysomes (disomes, trisomes, tetrasomes, etc). The monosome peak corresponds to both the free and mRNA-bound ribosomes, and the polysome peak corresponds to the mRNA bound ribosomes [31]. Polyribosome isolation [32] was carried out from growing as well as starved cells. Upon density gradient centrifugation, the expected profile in control consists of a peak corresponding to monosomes followed by several peaks corresponding to polysomes. Polysome profiles of all three types (Control, AX2:A15S4^+^ and AX2:A15S4^−^) resembled each other sufficiently for us to conclude that the effects of over- or under-expressing *DdS4* were unlikely to be caused by changes in ribosomal function at least with regard to gross translational efficiency (Figure 7A). We also carried out western blot analysis of the various fractions obtained after density gradient centrifugation to check where DdS4 primarily resides. We found equal representation of the DdS4 protein in the 40S, 60S and monosomes and the polysomes fraction when compared across control, AX2:A15S4^+^ and AX2:A15S4^−^ cells (Figure 7B). Interestingly, we also found DdS4 in the free protein fraction (not associated with the ribosomes). The free fraction isolated from AX2:A15S4^+^ contained more DdS4 as compared to controls while the fraction from AX2:A15S4^−^ contained a negligible amount of the protein (Figure 7B & 7C). This suggests that when over- or under expressed, only the free pool of the DdS4 protein is perturbed and the pool dedicated for ribosomes remains unaltered. We also carried out a S35 pulse chase experiment as an index of overall protein synthesis rates. Under conditions of *DdS4* over- and under-expression, AX2, AX2:A15S4^+^ and AX2:A15S4^−^ cells showed no discernible differences as indicated by three most prominent bands seen on the autoradiograph (Figure S3), indicating that overall protein turnover rates were unchanged.

### DdS4 over- and under-expressers display a dosage effect

As described earlier under- or over-expression of *DdS4* had similar phenotypic consequences during vegetative growth and development. Besides, in both cases the effects on the phenotype were graded similarly. Independent clones of AX2:A15S4^+^ and AX2:A15S4^−^ cells that were subjected to varying selection intensities showed differences in phenotype that were positively correlated with the concentration of antibiotic used (Figure 7D and Table 6). Three independent clones falling under each class (over- or under-expresser), labelled ‘low’, ‘medium’ and ‘high’, were monitored. ‘Low’ cells were designated with the suffix ‘l’ as AX2:A15S4^+^_l_ and AX2:A15S4^−^_l_ depending on whether they carried the DdS4 in the sense (+) or antisense (−) orientation; ‘medium’ and ‘high’ cells were designated similarly but with the suffixes m and h. ‘Low’ cells (AX2:A15S4^+^_l_ and AX2:A15S4^−^_l_) could grow on no more than 5 µg/ml of neomycin sulphate, ‘medium’ cells (AX2:A15S4^+^_m_ and AX2:A15S4^−^_m_) tolerated 10 µg/ml and ‘high’ cells (AX2:A15S4^+^_h_ and AX2:A15S4^−^_h_), up to 15 µg/ml. Western blots using antibodies to DdS4 confirmed that the levels of S4 protein in the low, medium and high classes differed as expected in both sense and antisense transformants (Figure 7E). During vegetative growth, the clones showed concentration-dependent differences in doubling times (Table 6). During development, all clones reproduced the two traits reported in the earlier section: aggregation streams broke up and aggregation territory sizes were significantly smaller than usual. The terminal phenotype of ‘low’ cells consisted of a misshapen spore mass atop a short stalk with a broad base; ‘medium’ cells formed short club-shaped terminal structures with thickened stalks and a misshapen spore mass; and the development of ‘high’ cells ended development as finger-like erect structures with no discernible sori (Figure 7D). Additionally, the classes differed with regard to spore formation efficiency and spore viability (Table 6).

**Table 6:** The copy-number effect of DdS4 perturbation on spore formation, spore viability and doubling time.

	AX2:A15S4^+^ (Over-expressors)			AX2:A15S4^−^ (Under-expressors)		
	Low	Medium	High	Low	Medium	High
**Spore forming efficiency**	11±0.2%	8±1.5%	5±2.5%	15±0.8%	9±0.5%	4±0.2%
**Spore viability**	9±0.5%	5±0.75%	3±0.3%	7±0.8%	4±0.2%	3±0.4%
**Doubling time (hrs.)**	4.2±0.5	3.9±1.75	3.5±1.2	5.3±1.8	4.3±2	3.9±1.3

Quantitative differences amongst the DdS4 over- and under-expressors in terms of spore forming efficiency, spore viability (estimated on the basis of counting about 5000 spores per experiment for each strain. Mean ± S.D., N = 4) and doubling time (Mean ± S.D., N = 4).

The phenotypic effects seen by graded over and under expression of DdS4 may be because of the participation of DdS4 in a multi-protein complex. A simple application of the law of mass action shows that the imbalance caused by raising or lowering the concentration of any component of the complex can lead to similar consequences [33], [34]. DdS4 is known to be a component of the ribosome, a multi-subunit RNA-protein complex; however, polyribosome profiles ruled out the possibility that what we were seeing was a consequence of ribosomal malfunction. These observations confirmed our hypothesis that effects produced by DdS4 over- or under-expression are caused by its participation in the actin cytoskeleton remodelling complex.

**Figure 8:**
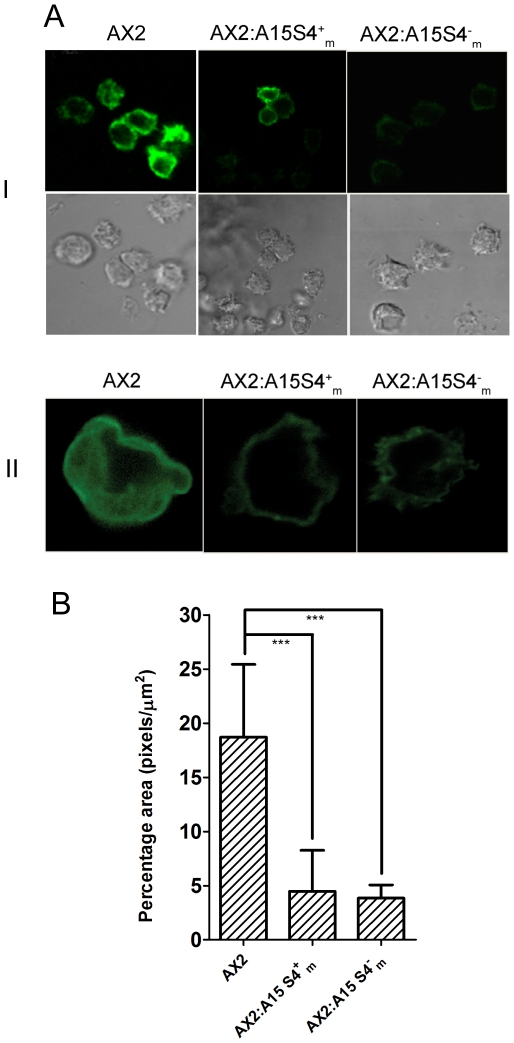
Cytoskeletal remodelling seen by actin-phalloidin staining. (**A**) Aggregating *D. discoideum* cells (AX2, AX2:A15S4^+^_m_ and AX2:A15S4^−^_m_). (**I**) Cells at 60× magnification with images at the top taken with green filter; corresponding bright field images are in the panels below. (**II**) Image of a typical cell after 3-D stacking. (**B**) The histograms indicate the mean phalloidin fluorescence intensity (calculated per µm^2^ of cells using Image J software). The p values were calculated using one-way ANOVA and were <0.05.

Budding in yeast involves a remodelling of the actin cytoskeleton [35]. Do the consequences of over- or under- expressing DdS4 in *D. discoideum* also impinge on the cytoskeleton? Initial observations suggest that they do. When we stained *D. discoideum* cells with Alexa Fluor 488-conjugated phalloidin, the fluorescently-labeled phalloidin decorated cytoskeletal actin as expected (Figure 8A) [36]. However, the staining was significantly more intense in the case of AX2 cells than with those of AX2:A15S4^+^_m_ or AX2:A15S4^−^_m_ (Figure 8B).

## Discussion

A foreign protein, DdS4, can rescue a set of yeast mutants (*cdc24*, *cdc42* and *bem1*) that are unable to proceed through the cell cycle, but ScS4 cannot. Consistent with its being a component of the ribosome, the attempt to isolate a loss-of-function mutation in *DdS4* was not successful. Indeed ribosomal function was buffered to such an extent that it appeared unaltered in *D. discoideum* amoebae in which DdS4 levels were raised or lowered via transformation with *DdS4* in a sense or antisense orientation (Figure 7A). However, the cells displayed characteristic morphogenetic defects (Figure 6 & Figure 7). The implication is that the defects were due to one or more ‘non-ribosomal’ functions of DdS4 being compromised. Eukaryotic ribosomal protein-encoding genes are known to have pleiotropic roles. They are associated with transcription, splicing, translation and DNA repair; their deficiency has been linked to developmental disorders in humans, fruit flies and plants [37], [38], [39]. In fish, ribosomal protein genes act as haplo-insufficient tumour suppressors and are likely to be involved in regulating normal development [40]. Interestingly, a knock-down mutation of the zebrafish *S4* gene leads to developmental defects, predominantly of neurological origin [41]. S19 deficiency in zebrafish leads to hematopoietic and developmental abnormalities due to dysregulation of delta Np63 and p53 [39], [42]. L13a, a ribosomal protein binds to the 3′-UTR of ceruloplasmin mRNA and inhibits its translation - but only after it has been phosphorylated and released from the ribosome [39]. Of particular interest to our study, RPL41, a ribosomal large subunit protein, associates with several cytoskeleton components including tubulin β, Y and myosin IIA [43]. In the case of *D. discoideum*, it has been reported that S4 binds inositol 6-phosphate [16]. The implications remain unknown, but the finding suggests that DdS4 also has a role in the plasma membrane or in signal transduction.

DdS4 is rather different from ScS4 in sequence; the latter groups with other fungal S4 proteins (Figure S4A & S4B). Sequence analysis points to similar domain architectures; a largely conserved N-terminus region and significant variations in the C-terminal end (Figure S4A & S4B). Some residues are conserved between ScBem1p homologues and DdS4 but vary between DdS4 and other S4's. These features explain why DdS4, but not ScS4, can substitute for the absence of one of the proteins in the bud site selection complex in yeast. Since DdS4 can compensate for the lack of ScCdc24p, ScCdc42p or ScBem1p in yeast, one possibility is that DdS4 could substitute for any of these proteins in the complex (Figure 1). However, this is functionally difficult to envisage. On the other hand, completely unrelated proteins can substitute for a scaffold protein since scaffolding functions are flexible and promiscuous [44]. Given that, we conjecture that in spite of significant differences in the amino acid sequences overall, DdS4 can act as a surrogate for ScBem1p; and the rescue by *DdS4* of *Sccdc24-4* and *Sccdc42* is a consequence of DdS4 functioning as a scaffold protein in yeast. Being temperature-sensitive, *Sccdc24-4* and *Sccdc42-1* are likely to be missense mutations. Though present in the cell at the restrictive temperature, the corresponding proteins will be unable to function normally because most molecules have an inappropriate conformation. When over-expressed, a surrogate scaffold protein can bind the misfolded protein and permit a functional complex to form. Alternatively, the surrogate could help to efficiently mobilise any residual properly folded protein and so restore normal function.

The developmental defects caused by expressing *DdS4* in antisense (AX2:A15S4^−^) or sense (AX2:A15S4^+^) orientations under a constitutive promoter were comparable and graded in parallel with the level of expression (Table 6). This finding strengthens the conjecture that DdS4 affects morphogenesis in *D. discoideum* by functioning as part of a multi-protein complex. Specifically, we were led to the balance hypothesis, which relates to a stoichiometry-related effect on the equilibrium concentration of the complex. The hypothesis is that when similar phenotypic defects result from under- or over-expression of a protein, the protein may be functioning as a component of a multimeric complex. In such a situation the imbalance caused by increasing or decreasing the concentration of a component of the complex can be similar and therefore the consequences can be similar too [33], [34], [45]. In our context, a change in the cellular level of total DdS4 in either direction has similar consequences because DdS4 functions as part of a multi-subunit protein complex that is analogous to the bud site selection complex in yeast.

Even though there is nothing like ‘bud site selection’ in *D. discoideum*, extensive cytoskeletal rearrangements are involved in morphogenesis [46]. On the basis of sequence similarity, *D. discoideum* has at least 16 Rac GTPases that resemble ScCdc42p and at least 10 proteins that appear to possess guanine nucleotide exchange activity that resemble ScCdc24p [47] (www.dictybase.org). Rac GTPases have been studied extensively in *D discoideum* and play unique roles in regulating actin polymerisation [47], [48], [49]. RacB, in particular, is of interest since its loss recapitulates some of the phenotypes that we report in this study, namely aggregate break up and abnormal terminal structures [50]. We note that the varied consequences of DdS4 under- or over-expression (on cell size, spore shape and multicellular morphology) could all be due to a defective cytoskeleton. How might DdS4 interact with ScCdc24 and ScCdc42? The presence of a domain similar to the SH3 domain in the C-terminus of Bem1p (Figure 4) may enable DdS4 to interact with Cdc42 in a manner analogous to ScBem1p. Sequence analysis also indicates that the probable mode of interaction between DdS4 and Cdc24 is via the SH3-like domain in the C-terminus (Figure S1D). In support of this, it turns out that *D. discoideum* sequences that resemble ScCdc24 (and contain CH, PH and RhoGEF domains) do have proline-rich stretches which could be potential sites for binding to an SH3 domain (Figure S1D).

Relative to normal development in AX2 cells, we observe a number of differences when DdS4 is under- or over-expressed. They include a reduction in cell size, faster cell division, smaller aggregates, aberrant-looking fruiting bodies, fewer, rounded and less viable spores. Each of the differences has been reported in some mutant of D. discoideum (see http://www.dictybase.org/db/cgi-bin/dictyBase/phenotype), but no mutant phenotype includes all of them. Similar to our findings (Figure 8A & 8B), the actin cytoskeleton is implicated in at least two cases. PIR121, Nap1, Abi2, HSPC300 and SCAR/WAVE form a multi-protein complex that drives actin polymerisation and cytoskeletal organisation. Cells lacking Nap1 [51] or *abiA* activity [52] are reduced in size. Fimbrin and ABP34 are two proteins that are involved in organising actin filaments into bundles. A mutant that lacks both shows a reduced cell size, small fruiting bodies and poor spore formation [53]. Not unexpectedly, these genes bear no obvious relation to *DdS4*, providing yet another demonstration of the truism that most of the time one cannot reason backwards from a change in the phenotype – however sharply defined – to its likely genetic basis.

Wright used the phrase ‘almost universal pleiotropy [54]’ to describe the observation that a change in the activity of a single gene usually affects many traits. Beyond the demonstration of pleiotropic roles for what had been characterised solely as a ribosomal protein, there is an interesting evolutionary implication of this work. Pleiotropy or ‘moonlighting’ is a pervasive feature of proteins [55], [56]. However, the roles played by DdS4 in the two multi-protein complexes of which it forms a part are not comparable in their importance for the organism. One role (as part of the ribosome) is essential for cell viability and requires tight regulation of the amount of DdS4 in a cell. On the other hand, the other role (as part of the putative complex discussed above) can accommodate substantial variations in DdS4 levels (morphogenesis is affected and sporulation efficiency is lowered in the transformants, but not all the way to zero; Table 2). The presence of a second role for the same protein means that its basal levels are higher than they would have been in its absence. The fact that variations in the level are more or less tolerated with regard to the second role means that the performance of the first role is buffered against mutationally- or environmentally-induced changes that might have been lethal otherwise. In our case, the second role acts as a built-in safeguard against the potentially lethal consequences of sub-optimal ribosomal activity that might be caused by spontaneous variations in DdS4 levels. This observation adds to the list of selective advantages for the evolution of multifunctionality in proteins [57].

## Materials and Methods

Unless stated otherwise, all chemicals were purchased from HiMedia, India or Difco, USA and are of analytical grade.

### Cell culture and development

*Dictyostelium* strains were grown on Phosphate buffer (15 mM KH_2_PO_4_, 2 mM K_2_PO_4_, pH 6.4) agar plates with *Klebsiella aerogenes* at 22°C. Neomycin resistant transformants were maintained with bacteria on PBA agar plates containing 12 µg/ml of neomycin. The number of viable spores was determined by harvesting all cells after 4 days of development, heating the cell suspension to 45°C for 5 minutes followed by treatment with 0.1% NP-40 for 10 minutes and counting the number of resistant cells that form plaques on bacterial lawns. For growth rate measurements, the DdS4 over- and under-expressors were grown with bacteria on agar plates containing appropriate antibiotic concentrations.

### Molecular biology

The DdS4 gene was amplified from AX2 genomic DNA using primers, 5′CAGGATCCAAGATGGCTCGTGGTCCA3′ and 5′CTCTAGAATTATTTATTTAAGCAACG3′. The gene was then cloned in pACT15XPT1 vector (a kind gift from Dr. David Knecht) to get DdS4 both in sense and antisense orientations with respect to the Actin 15 promoter. The constructs were then electroporated into AX2 to obtain AX2:A15S4^+^ and AX2:A15S4^−^. For generating the promoter-GFP construct, the putative promoter was amplified from AX2 genomic DNA with primers, 5′TTCTCGAGTAGACCACCCTAATGGTT3′ and 5′AATCTAGATGTTTCTTTGGACCACGA3′ and sub cloned into pDRIVE vector (Qiagen TA cloning kit) and subsequently into pREMIGFP vector.

A single primer, 5′ GTC AGA CGT TCA AGA GTC GAC GAA CGT GAC GCT GCC CTC AAG AGA 3′, was used to generate single amino acid substitution (N253D) in DdS4. To generate a deletion of 13 amino acids in DdS4, 5′ATGCATGCTACGTATAGGTGAAAAAATCGAAACCG 3′ and 5′ ATGCATGCTCTACGTACACCTTTACCGGCTGGGAG 3′ primers were used.

*Saccharomyces cerevisiae* transformation was done by the Lithium acetate- Poly ethylene glycol method [58]. For complementation studies, DdS4 and ScS4 (using primers 5′CGGATCCATGGCTAGAGGACCAAAGAAGCATCTAAAGAGA3′ and 5′ CCTCGAGTTACAAACCTTGTTGAGCT 3′) were cloned in pYES2. Temperature shift was used to synchronise *cdc* mutants of *Saccharomyces cerevisiae*. Briefly cells were grown to a density of 10^6^/ml in YPD at 30°C under shaking conditions using an overnight grown culture as inoculum. The culture was then shifted to a shaker maintained at 37°C and incubated for a period of two and half hours to arrest the entire population in a cell cycle specific manner. The arrest was confirmed by FACS analysis. Cell cycle by FACS was carried out following the method described by Hutter and Eipel [59]. The λADH library obtained was amplified following the method described by Miller to obtain a titre of 10^11^ pfu/ml [60]. For translational fidelity assay, strains with different auxotrophic or cell cycle mutation either due to missense and nonsense mutations were used. In brief, these were transformed with the plasmid encoding the ribosomal protein and the untransformed and the transformed cells were plated on auxotrophic media and maintained under selective conditions. The number of colonies appearing under each condition were analysed after 72 hours and this was used to calculate the translational error rate, a function inverse to translational fidelity, with the wild type error rate as a background control.

*bem1* knockout in haploid *S. cerevisiae* was created by amplifying the Leu cassette from pRS405 using the primers 5′ GTTTTCACTAATATACTAAACCCATATGGATGCACGTTGAAAGCACTGTGTGAAAAGAAAGGATAATTATACTCTATT 3′and 5′ GAATCTTATTACTCTCGCAAATCATCATAAAGTTGATTAAAATGTTCAAC CTATGTGCAT TTTCTATTATGAATTTCA 3′. The wild type yeast was transformed with either DdS4 or yeast *bem1* (amplified using primers 5′ ATGCGGATCCATGCTGAAAAACTTC 3′ and 5′ GATCCTCGAGTCAAATATCGTGAAC 3′ and cloned in pYES2 vector). The untransformed yeast strain was the control. Colonies that grew on the minimal plate containing URA3 were chosen for transformation with the linear DNA (the PCR product). The colonies that grew on LEU2- URA3- plates at 30°C were selected. The clones are confirmed using the primers 5′ TGGCGAAGGGCCCCATTCAT 3′ and 5′GCGTCAGGCGACCTCTGAAA 3′. Since the forward primer binds within a region flanking the *bem1* gene at the chromosomal locus, a correct sized amplicon of ∼400 bps will be seen only if the knockout cassette has inserted at the correct location on the chromosome.

### Antibody generation and Western blot analysis

Full length coding region of DdS4 was cloned in pET16b and was transformed into *E. coli* BL21.The His- protein was then used for generation of polyclonal antibodies in rabbit.

For Western blot analysis, cells were harvested at various developmental stages, washed, solubilized in lysis buffer [100 mM Nacl, 5 mM EDTA,50 Mm Tris pH 7.5, 1× protease inhibitor cocktail (Roche)], and freeze thawed 3–4 times. Equal amount of cellular proteins (as determined by Bradford assay) were loaded on a 12% polyacrylamide SDS gel and subsequently transferred to PVDF membrane. After blocking, the blots were incubated for 3 hrs with the primary anti–DdS4 antibody at 1∶2000 dilution, washing 3 times with PBS-T, and incubating with secondary anti rabbit goat antibody (Millipore) at 1∶2000 dilution. After washes, the bound antibody was detected with an ECL Western blotting kit (Perkin Elmer). For commercial antibodies, anti-CDC24 (MCF2/Dbl antibody, Cell Signaling Technology) and anti-CDC42 (Cell Signaling Technology) dilution used was 1∶1000 and the buffer used was TBS-T.

*E. coli* BL21 over-expressing Cdc42p-GST, Cdc24p-GST, Bem1p-GST and Cla4p-GST proteins (the constructs were kind gift from Dr D. Lew) were harvested and washed with lysis buffer. After pelleting the cells the supernatant was used for interaction with GSH beads for 1 hr in a rocker at 4°C. For specific interaction, the beads were incubated with the supernatant containing the over-expressed protein. This was incubated on a rocker at 4°C for 2 hrs. The beads are then washed with interaction buffer [50 mM HEPES, 100 mM Nacl, 1× Triton X. 1 mM DTT, 5 mM EDTA, IX protease inhibitor Cocktail (Roche)] followed by a PBS wash. The beads are then boiled in the SDS loading dye or in loading dye without β-mercaptoethanol for blots where anti-CDC42 was used and loaded on a 12% SDS-PAGE. The commercial antibodies used for identifying DdCdc24 and DdCdc42 were raised against the human MCF2/Dbl which has ∼69% similarity with full length ScCdc24p and the residues surrounding Lys135 of human Cdc42 respectively which is known to be crucial for the GTPase activity of Cdc42 in several species including *S. cerevisiae*.

### Co-immunoprecipitation studies

For co-immunoprecipitation, the supernatants of the over-expressing clones upon lysis with glass beads [425–600 microns (Sigma Chemicals, USA)] were pooled. This cocktail was subjected to preclearing with 10 µg pre-immune serum IgG and Sepharose-protein G (Sigma Chemicals, USA) slurry on a rocker at 4°C for 3 hrs. DdS4 protein antibody was added and incubated overnight at 4°C on the rocker. Subsequently sepharose-protein G beads were added and allowed to interact for 2 hrs. The pellet was then loaded after boiling on SDS-PAGE.

### Immunohistochemical staining

For actin phalloidin staining, AX2 cells were fixed with 4% paraformaldehyde for 30 mins at room temperature [61]. Washes with TBS-T (0.1% Triton-X in TBS) were followed by blocking with 5% milk for half an hour. Rhobdamine-phalloidin staining (1∶375 dilution) (Molecular Probes) at 37°C for 65 mins was carried out. Three washes with TBS-T were given and cells were mounted in vectamount (Vector Laboratories) on slides and viewed in confocal microscope.

### RNA in situ Hybridisation

The DNA probe (1–3 µg) was labelled for 20 hrs by random priming by incorporation of dUTP-digoxigenin using the ‘DIG High prime’ labelling kit from Roche Molecular Biochemicals, Germany (cat. no. 1 585 606) according to the manufacturer's instructions. Probes were purified by ethanol precipitation. The protocol used for hybridization, washing and color development was essentially as described by Escalante and Loomis (1995) with minor modifications. For separation of prestalk and prespore cells, slugs were harvested and cells were disaggregated in KK2 buffer containing 20 mM EDTA. A gradient of 70% Percoll (Sigma, USA) in KK2 buffer containing 20 mM EDTA was made in 15 ml Corex tube at 27,200 g for 40 mins.10^8^ cells in 250 µl was layered on this gradient and spun at 17,400 g for 3 mins. The cells were recovered by washing them with KK2 buffer. For subcellular fractionation the procedure has been described previously [23].

### Polysome assay

Amoebae were harvested from nutrient medium at ∼5×10^6^–10^7^ cells/ml by centrifuging at 300 g for 3 mins and immediately chilled on ice. Cells were resuspended in buffer followed by addition of cycloheximide at a concentration of 150 µg/ml and allowed to incubate on ice for additional 5 mins. The cells are spun at 4000 rpm for 5 mins in cold. The cells are washed once with lysis buffer (50 mM HEPES pH 7.5, 5 mM MgCl_2_, 40 mM Magnesium acetate, 20 mM potassium phosphate, 0.5 mM DTT, 0.1 mg/ml cycloheximide, 5% sucrose and 0.4% NP-40) and subsequently the pellet is frozen in liquid nitrogen. 200 µl of lysis buffer and an equal volume of glass beads were added to the pellet. This is vortexed at full speed for 2 mins followed by incubation in ice for 5 mins. Repeat this thrice. Spin down the cells at 4000 rpm for 2 mins in cold. The concentration of RNA was determined spectrophotometrically and 20 units of RNA was layered onto 11 ml of 10–50% linear sucrose gradient. Centrifugation was carried out at 28000 rpm for 4 hrs at 4°C. Gradients were fractionated by upward displacement in a gradient fractionator equipped with a UV monitor.

For S^35^ labelling, 1×10^8^cells were spotted on filter paper and allowed to stand for 15 mins. The filter paper was then placed on 50 microlitre droplets of 750 µCi label (Perkin Elmer) in an empty petridish and incubated for an hour. The filter paper was placed in an eppendorf and spun down to collect the cells. The pellet was resuspended in buffer without the label and frozen. This was time t = 0. For later time points, the cells in buffer were plated on SM/5 plates with bacteria and collected at times indicated.

### Computational sequence analysis and structure prediction

Sequences of ScBem1p, DdS4, ScS4 and their related proteins were been obtained from Uniprot [62]. Domain assignments based on functional similarity was obtained from Pfam [26] family database by doing a HMM based search in the protein family database. Structural domain assignments were obtained by using 3D-Jigzaw [63], an automated server for comparative modeling and Superfmaily database system [64] which queries the sequence against SCOP [65] families and superfamilies. Subsequent to identification of the domain boundaries, sequence fragments encompassing the domain and variable flanking regions were analysed using 3 d Jury, a metaserver [66] which provides consensus based structure prediction. Such an approach has higher accuracy than individual structure prediction algorithms. From the server a consensus base secondary structure prediction was obtained. Using the server structural fold was predicted for the submitted sequence by different approaches such as mgenthreader [67], FUGUE [68]. Independently the sequences were also submitted to phyre [69] for fold prediction. An appropriate structural template was chosen based on consensus from highly significant and reliable hits obtained from different fold prediction methods. Structural modeling and modeled structure validation was done using modeler version 9 [70] and PROSA [71] respectively. In order to obtain functionally critical residues conserved across other homologues sequences CONSURF [72] was used. CONSURF takes up a structure as an input extracts its sequence and searches for its homologs (e-value below 0.0001; database uniprot). These homologous sequences are then aligned {MUSCLE [73]} and residue-wise conservation is obtained using Bayesian method. To obtain locally similar regions between ScBem1p and DdS4, MEME [74] was used. MEME detects conserved, statistically significant local patterns (motifs) amongst a group of sequences. In order to identify SH3 binding regions in DdCdc24 SH3 hunter [75] was used.

## Supporting Information

Figure S1 **Structural insights into DdS4 interactions.** (**A**) Domain organization of ScBem1p (**I**) and DdS4p (**II**). SCOP superfamilies were obtained from Superfamily.org database [77]. Definitions for SCOP ids are as follows: **b.34.2** : All Beta protein, SH3 like barrel, SH3 domain; **b.34.5** : All Beta, SH3-like barrel, Translation proteins SH3- like domain; **d.189.1** -: Alpha and beta proteins, PX domain; **d.15.2** -: Alpha and beta proteins, beta-Grasp(ubiquitin like), CAD & PB1 domain. (**B**) Consensus based secondary structure prediction of DdS4. C-terminal region, c = coil, e = extended sheet, h = helix. Gray shaded box depicts the model for the DdS4 C-terminal region. The color coding of the structure is based on the extent of conservation of residues amongst the S4 homologs (as obtained from ConSurf). Blue colored regions of the structure are most conserved, while red colored regions are least conserved . (**C**) JOY alignment [78] between the template structure 1JJ2 [79] and the c-terminal region of S4 (150 residue onwards). Protein bank id 1JJ2 corresponds to the structure of the protein encoding the Large Ribosomal Subunit from *Haloarcula marismortui*. The S chain from this structure was used for modelling the C-termini of S4. This chain is annotated as 50S ribosomal protein L24P and bears structural resemblance to SH3. Different secondary structural elements of 1JJ2 are labeled as helix (a), Sheets (b) and 3_10_ helix (3). Solvent accessible residues are represented in lower case, solvent inaccessible residues in upper case, residues hydrogen bonded to main-chain amide are in **bold**, residues hydrogen bonded to main-chain carbonyl are underlined and positive phi torsion angle are represented in *italic*. (**D**) PXXPX motifs in Cdc24 proteins from *Dictyostelium*. Regions in bold depict proline rich regions in Cdc24 proteins form *Dictyostelium* as obtained from uniprot. PXXPX is involved in recognition and binding to SH3 domain. Regions with high significance score are highlighted in red (significance score obtained from SH3 hunter) [75].
(TIF)

Figure S2 **Spatial localization** ***DdS4*** **mRNA, anti-DdS4 antibody specificity and sub-cellular localization of DdS4.** (**A**) In situ hybridisation of developmental stages using a DdS4 DNA probe. (**I**) Tipped mound (**II**) Slug (**III**) Early culminant (**IV**) control using H3a DNA probe. (**B**) Specificity of anti-DdS4 antibody was determined by western blot using (**I**) E. coli BL21 lysate and probing with anti-His antibody (DdS4 is expressed as a His-tag protein) and (**II**) *D. discoideum* lysates where increasing amounts of lysates were loaded from either control AX2 or AX2::A15S4^+^. (**III**) Specific interactions between the GST-tagged proteins and DdS4 are observed in the IP lane. Refer Figure 2A legend for details. This is an over-exposed blot to visualize pull downs of Bem1p and Cla4p more clearly. (**C**) Subcellular fractionation of cells. Aggregating AX2 cells were subjected to high speed centrifugation to yield cytosolic and membrane fractions. The soluble and insoluble cytoskeletal fractions were also checked for DdS4. Equal loading was checked by Coomassie staining.
(TIF)

Figure S3 **AX2:A15 mutants display normal protein synthesis.** DdS4 cells were pulse-labelled for 1 h with [^35^S]methionine and chased for 4 h. The protein bands indicate equal rate of incorporation and subsequent chase of ^35^S label.
(TIF)

Figure S4 **DdS4 is different from ScS4 at the C-terminal end.** (**A**) Motifs conserved across S4 homologs (**I**). (**II**) The C-terminal region from few DdS4 homologs (*S.cerevisiae, P.falciparum, S.pombe, L.elongisporus, C.cinerea okayama*) depicting motif 11 (purple), 15 (yellow) and 16 (blue). S4 homolgs were submitted in MEME [80] to identify locally conserved regions (depicted by different colored rectangular boxes numbered according to the e-value, 1 showing the lowest e-value). N-terminus is largely conserved while variation largely lies in the C-terminus. DdS4 C-terminus lacks conserved motifs 15 and 16 ; purple region depicts motif 11 while yellow and blue regions show motifs 15 and 16 respectively. Motif 15 and 16 are not conserved in DdS4 showing a very high e-value. (**B**) Tree depicting all the S4 homologous sequences. All sequences considered are of eukaryotic origin. Tree was generated using Neighborhood joining method and validated by bootstrapping for 500 iterations (values indicated on the branches). Nodes have been clustered and color coded based on the kingdom, all the Metazoan sequences are represented in Purple, Plants in green and fungi in Blue. ScS4 (Dark Blue edge) clusters with other fungal S4 sequences (sky blue edge), DdS4 (dark blue edge) shows variation from other S4 (more similar to Plants). Tree generation and statistical testing done using MEGA [81]
(TIF)

## References

[1] Bonner JT (1967). The cellular slime molds.

[2] Raper KB, Rahn AW (1984). The dictyostelids.

[3] Leach CK, Ashworth JM, Garrod DR (1973). Cell sorting out during the differentiation of mixtures of metabolically distinct populations of Dictyostelium discoideum.. J Embryol Exp Morphol.

[4] McDonald SA, Durston AJ (1984). The cell cycle and sorting behaviour in Dictyostelium discoideum.. J Cell Sci.

[5] Saran S, Azhar M, Manogaran PS, Pande G, Nanjundiah V (1994). The level of sequestered calcium in vegetative amoebae of Dictyostelium discoideum can predict post-aggregative cell fate.. Differentiation.

[6] Azhar M, Kennady PK, Pande G, Espiritu M, Holloman W (2001). Cell cycle phase, cellular Ca2+ and development in Dictyostelium discoideum.. Int J Dev Biol.

[7] Beach D, Durkacz B, Nurse P (1982). Functionally homologous cell cycle control genes in budding and fission yeast.. Nature.

[8] Michaelis C, Weeks G (1993). The isolation from a unicellular organism, Dictyostelium discoideum, of a highly-related cdc2 gene with characteristics of the PCTAIRE subfamily.. Biochim Biophys Acta.

[9] Souza GM, Lu S, Kuspa A (1998). YakA, a protein kinase required for the transition from growth to development in Dictyostelium.. Development.

[10] Fields SD, Conrad MN, Clarke M (1998). The S. cerevisiae CLU1 and D. discoideum cluA genes are functional homologues that influence mitochondrial morphology and distribution.. J Cell Sci.

[11] Butty AC, Perrinjaquet N, Petit A, Jaquenoud M, Segall JE (2002). A positive feedback loop stabilizes the guanine-nucleotide exchange factor Cdc24 at sites of polarization.. EMBO J.

[12] Casamayor A, Snyder M (2002). Bud-site selection and cell polarity in budding yeast.. Curr Opin Microbiol.

[13] Irazoqui JE, Lew DJ (2004). Polarity establishment in yeast.. J Cell Sci.

[14] Anand S, Prasad R (1987). Status of calcium influx in cell cycle of S. cerevisiae.. Biochem Int.

[15] Sloat BF, Adams A, Pringle JR (1981). Roles of the CDC24 gene product in cellular morphogenesis during the Saccharomyces cerevisiae cell cycle.. J Cell Biol.

[16] Tapparo A, Satre M, Klein G (1998). Cloning, sequencing and developmental expression of the genes encoding S4 and S10 ribosomal proteins in the cellular slime mould Dictyostelium discoideum.. Curr Genet.

[17] Corney AJ, Richards AJ, Phillpots T, Hames BD (1990). Developmental regulation of cell-type-enriched mRNAs in Dictyostelium discoideum.. Mol Microbiol.

[18] Proffitt JA, Jagger PS, Wilson GA, Donovan JT, Widdowson DC (1991). A developmentally regulated gene encodes the dictyostelium homolog of yeast ribosomal protein S4 and mammalian LLRep3 proteins.. Nucleic Acids Res.

[19] Inazu Y, Chae SC, Maeda Y (1999). Transient expression of a mitochondrial gene cluster including rps4 is essential for the phase-shift of Dictyostelium cells from growth to differentiation.. Dev Genet.

[20] Atkins JF, Elseviers D, Gorini L (1972). Low activity of -galactosidase in frameshift mutants of Escherichia coli.. Proc Natl Acad Sci U S A.

[21] Stansfield I, Jones KM, Herbert P, Lewendon A, Shaw WV (1998). Missense translation errors in Saccharomyces cerevisiae.. J Mol Biol.

[22] Bose I, Irazoqui JE, Moskow JJ, Bardes ES, Zyla TR (2001). Assembly of scaffold-mediated complexes containing Cdc42p, the exchange factor Cdc24p, and the effector Cla4p required for cell cycle-regulated phosphorylation of Cdc24p.. J Biol Chem.

[23] Mondal S, Neelamegan D, Rivero F, Noegel AA (2007). GxcDD, a putative RacGEF, is involved in Dictyostelium development.. BMC Cell Biol.

[24] Moskow JJ, Gladfelter AS, Lamson RE, Pryciak PM, Lew DJ (2000). Role of Cdc42p in pheromone-stimulated signal transduction in Saccharomyces cerevisiae.. Mol Cell Biol.

[25] Giaever G, Chu AM, Ni L, Connelly C, Riles L (2002). Functional profiling of the Saccharomyces cerevisiae genome.. Nature.

[26] Finn RD, Tate J, Mistry J, Coggill PC, Sammut SJ (2008). The Pfam protein families database.. Nucleic Acids Res.

[27] Maiti R, Van Domselaar GH, Zhang H, Wishart DS (2004). SuperPose: a simple server for sophisticated structural superposition.. Nucleic Acids Res.

[28] Yamaguchi Y, Ota K, Ito T (2007). A novel Cdc42-interacting domain of the yeast polarity establishment protein Bem1. Implications for modulation of mating pheromone signaling.. J Biol Chem.

[29] Nakai K, Horton P (1999). PSORT: a program for detecting sorting signals in proteins and predicting their subcellular localization.. Trends Biochem Sci.

[30] Knecht DA, Cohen SM, Loomis WF, Lodish HF (1986). Developmental regulation of Dictyostelium discoideum actin gene fusions carried on low-copy and high-copy transformation vectors.. Mol Cell Biol.

[31] Hirashima A, Kaji A (1972). Factor-dependent release of ribosomes from messenger RNA. Requirement for two heat-stable factors.. J Mol Biol.

[32] Arava Y, Wang Y, Storey JD, Liu CL, Brown PO (2003). Genome-wide analysis of mRNA translation profiles in Saccharomyces cerevisiae.. Proc Natl Acad Sci U S A.

[33] Papp B, Pal C, Hurst LD (2003). Dosage sensitivity and the evolution of gene families in yeast.. Nature.

[34] Veitia RA (2003). Nonlinear effects in macromolecular assembly and dosage sensitivity.. J Theor Biol.

[35] Hall A (1994). Small GTP-binding proteins and the regulation of the actin cytoskeleton.. Annu Rev Cell Biol.

[36] Lee E, Knecht DA (2001). Cytoskeletal alterations in Dictyostelium induced by expression of human cdc42.. Eur J Cell Biol.

[37] Wool IG (1996). Extraribosomal functions of ribosomal proteins.. Trends Biochem Sci.

[38] Podkovyrov S, Larson TJ (1995). Lipid biosynthetic genes and a ribosomal protein gene are cotranscribed.. FEBS Lett.

[39] Mazumder B, Sampath P, Seshadri V, Maitra RK, DiCorleto PE (2003). Regulated release of L13a from the 60S ribosomal subunit as a mechanism of transcript-specific translational control.. Cell.

[40] Amsterdam A, Sadler KC, Lai K, Farrington S, Bronson RT (2004). Many ribosomal protein genes are cancer genes in zebrafish.. PLoS Biol.

[41] Uechi T, Nakajima Y, Nakao A, Torihara H, Chakraborty A (2006). Ribosomal protein gene knockdown causes developmental defects in zebrafish.. PLoS One.

[42] Danilova N, Sakamoto KM, Lin S (2008). Ribosomal protein S19 deficiency in zebrafish leads to developmental abnormalities and defective erythropoiesis through activation of p53 protein family.. Blood.

[43] Wang S, Huang J, He J, Wang A, Xu S (2010). RPL41, a small ribosomal peptide deregulated in tumors, is essential for mitosis and centrosome integrity.. Neoplasia.

[44] Park SH, Zarrinpar A, Lim WA (2003). Rewiring MAP kinase pathways using alternative scaffold assembly mechanisms.. Science.

[45] Bender A, Pringle JR (1989). Multicopy suppression of the cdc24 budding defect in yeast by CDC42 and three newly identified genes including the ras-related gene RSR1.. Proc Natl Acad Sci U S A.

[46] Eichinger L, Lee SS, Schleicher M (1999). Dictyostelium as model system for studies of the actin cytoskeleton by molecular genetics.. Microsc Res Tech.

[47] Rivero F, Dislich H, Glockner G, Noegel AA (2001). The Dictyostelium discoideum family of Rho-related proteins.. Nucleic Acids Res.

[48] Para A, Krischke M, Merlot S, Shen Z, Oberholzer M (2009). Dictyostelium Dock180-related RacGEFs regulate the actin cytoskeleton during cell motility.. Mol Biol Cell.

[49] Wilkins A, Insall RH (2001). Small GTPases in Dictyostelium: lessons from a social amoeba.. Trends Genet.

[50] Park KC, Rivero F, Meili R, Lee S, Apone F (2004). Rac regulation of chemotaxis and morphogenesis in Dictyostelium.. EMBO J.

[51] Ibarra N, Blagg SL, Vazquez F, Insall RH (2006). Nap1 regulates Dictyostelium cell motility and adhesion through SCAR-dependent and -independent pathways.. Curr Biol.

[52] Pollitt AY, Insall RH (2008). Abi mutants in Dictyostelium reveal specific roles for the SCAR/WAVE complex in cytokinesis.. Curr Biol.

[53] Pikzack C, Prassler J, Furukawa R, Fechheimer M, Rivero F (2005). Role of calcium-dependent actin-bundling proteins: characterization of Dictyostelium mutants lacking fimbrin and the 34-kilodalton protein.. Cell Motil Cytoskeleton.

[54] Wright S (1964). Pleiotropy in the evolution of structural reduction and dominance.. Am Nat.

[55] Hodgkin J (1998). Seven types of pleiotropy.. Int J Dev Biol.

[56] Huberts DH, van der Klei IJ (2010). Moonlighting proteins: an intriguing mode of multitasking.. Biochim Biophys Acta.

[57] Erijman A, Aizner Y, Shifman JM (2011). Multispecific recognition: mechanism, evolution, and design.. Biochemistry.

[58] Klebe RJ, Harriss JV, Sharp ZD, Douglas MG (1983). A general method for polyethylene-glycol-induced genetic transformation of bacteria and yeast.. Gene.

[59] Hutter KJ, Eipel HE (1979). Microbial determinations by flow cytometry.. J Gen Microbiol.

[60] Miller JH (1992). A Short Course in Bacterial Genetics: A Laboratory Manual and Handbook for Escherichia coli and Related Bacteria.

[61] Watts DJ, Ashworth JM (1970). Growth of myxameobae of the cellular slime mould Dictyostelium discoideum in axenic culture.. Biochem J.

[62] Boutet E, Lieberherr D, Tognolli M, Schneider M, Bairoch A (2007). UniProtKB/Swiss-Prot.. Methods Mol Biol.

[63] Bates PA, Kelley LA, MacCallum RM, Sternberg MJ (2001). Enhancement of protein modeling by human intervention in applying the automatic programs 3D-JIGSAW and 3D-PSSM.. Proteins.

[64] Wilson D, Madera M, Vogel C, Chothia C, Gough J (2007). The SUPERFAMILY database in 2007: families and functions.. Nucleic Acids Res.

[65] Andreeva A, Howorth D, Chandonia JM, Brenner SE, Hubbard TJ (2008). Data growth and its impact on the SCOP database: new developments.. Nucleic Acids Res.

[66] Kajan L, Rychlewski L (2007). Evaluation of 3D-Jury on CASP7 models.. BMC Bioinformatics.

[67] Jones DT (1999). GenTHREADER: an efficient and reliable protein fold recognition method for genomic sequences.. J Mol Biol.

[68] Shi J, Blundell TL, Mizuguchi K (2001). FUGUE: sequence-structure homology recognition using environment-specific substitution tables and structure-dependent gap penalties.. J Mol Biol.

[69] Bennett-Lovsey RM, Herbert AD, Sternberg MJ, Kelley LA (2008). Exploring the extremes of sequence/structure space with ensemble fold recognition in the program Phyre.. Proteins.

[70] Eswar N, Eramian D, Webb B, Shen MY, Sali A (2008). Protein structure modeling with MODELLER.. Methods Mol Biol.

[71] Wiederstein M, Sippl MJ (2007). ProSA-web: interactive web service for the recognition of errors in three-dimensional structures of proteins.. Nucleic Acids Res.

[72] Goldenberg O, Erez E, Nimrod G, Ben-Tal N (2009). The ConSurf-DB: pre-calculated evolutionary conservation profiles of protein structures.. Nucleic Acids Res.

[73] Edgar RC (2004). MUSCLE: multiple sequence alignment with high accuracy and high throughput.. Nucleic Acids Res.

[74] Bailey TL, Williams N, Misleh C, Li WW (2006). MEME: discovering and analyzing DNA and protein sequence motifs.. Nucleic Acids Res.

[75] Ferraro E, Peluso D, Via A, Ausiello G, Helmer-Citterich M (2007). SH3-Hunter: discovery of SH3 domain interaction sites in proteins.. Nucleic Acids Res.

[76] Glaser F, Pupko T, Paz I, Bell RE, Bechor-Shental D (2003). ConSurf: identification of functional regions in proteins by surface-mapping of phylogenetic information.. Bioinformatics.

[77] Gough J, Karplus K, Hughey R, Chothia C (2001). Assignment of homology to genome sequences using a library of hidden Markov models that represent all proteins of known structure.. J Mol Biol.

[78] Mizuguchi K, Deane CM, Blundell TL, Johnson MS, Overington JP (1998). JOY: protein sequence-structure representation and analysis.. Bioinformatics.

[79] Klein DJ, Schmeing TM, Moore PB, Steitz TA (2001). The kink-turn: a new RNA secondary structure motif.. EMBO J.

[80] Bailey TL, Boden M, Buske FA, Frith M, Grant CE (2009). MEME SUITE: tools for motif discovery and searching.. Nucleic Acids Res.

[81] Kumar S, Nei M, Dudley J, Tamura K (2008). MEGA: a biologist-centric software for evolutionary analysis of DNA and protein sequences.. Brief Bioinform.

