# Supplementary figures and images for: Pleiotropic Roles of a Ribosomal Protein in Dictyostelium discoideum
Source: PLoS One. 2012 Feb 17;7(2):e30644. doi: 10.1371/journal.pone.0030644 (PMC3281849; doi:10.1371/journal.pone.0030644)

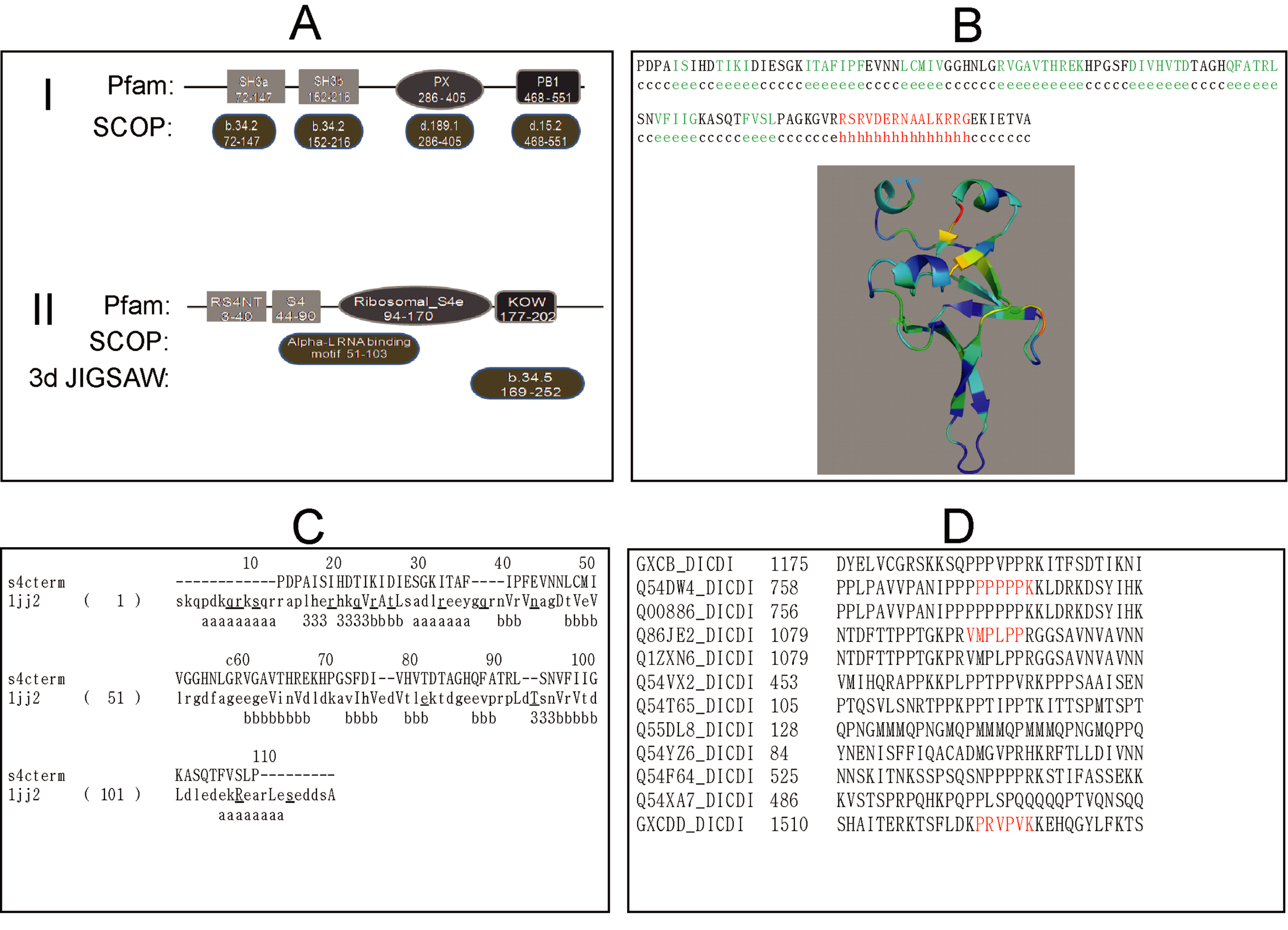

Supplement: Figure S1 — Structural insights into DdS4 interactions. (A) Domain organization of ScBem1p (I) and DdS4p (II). SCOP superfamilies were obtained from Superfamily.org database [77]. Definitions for SCOP ids are as follows: b.34.2 : All Beta protein, SH3 like barrel, SH3 domain; b.34.5 : All Beta, SH3-like barrel, Translation proteins SH3- like domain; d.189.1 -: Alpha and beta proteins, PX domain; d.15.2 -: Alpha and beta proteins, beta-Grasp(ubiquitin like), CAD & PB1 domain. (B) Consensus based secondary structure prediction of DdS4. C-terminal region, c = coil, e = extended sheet, h = helix. Gray shaded box depicts the model for the DdS4 C-terminal region. The color coding of the structure is based on the extent of conservation of residues amongst the S4 homologs (as obtained from ConSurf). Blue colored regions of the structure are most conserved, while red colored regions are least conserved . (C) JOY alignment [78] between the template structure 1JJ2 [79] and the c-terminal region of S4 (150 residue onwards). Protein bank id 1JJ2 corresponds to the structure of the protein encoding the Large Ribosomal Subunit from Haloarcula marismortui. The S chain from this structure was used for modelling the C-termini of S4. This chain is annotated as 50S ribosomal protein L24P and bears structural resemblance to SH3. Different secondary structural elements of 1JJ2 are labeled as helix (a), Sheets (b) and 310 helix (3). Solvent accessible residues are represented in lower case, solvent inaccessible residues in upper case, residues hydrogen bonded to main-chain amide are in bold, residues hydrogen bonded to main-chain carbonyl are underlined and positive phi torsion angle are represented in italic. (D) PXXPX motifs in Cdc24 proteins from Dictyostelium. Regions in bold depict proline rich regions in Cdc24 proteins form Dictyostelium as obtained from uniprot. PXXPX is involved in recognition and binding to SH3 domain. Regions with high significance score are highlighted in red (significance [file pone.0030644.s001.tif]

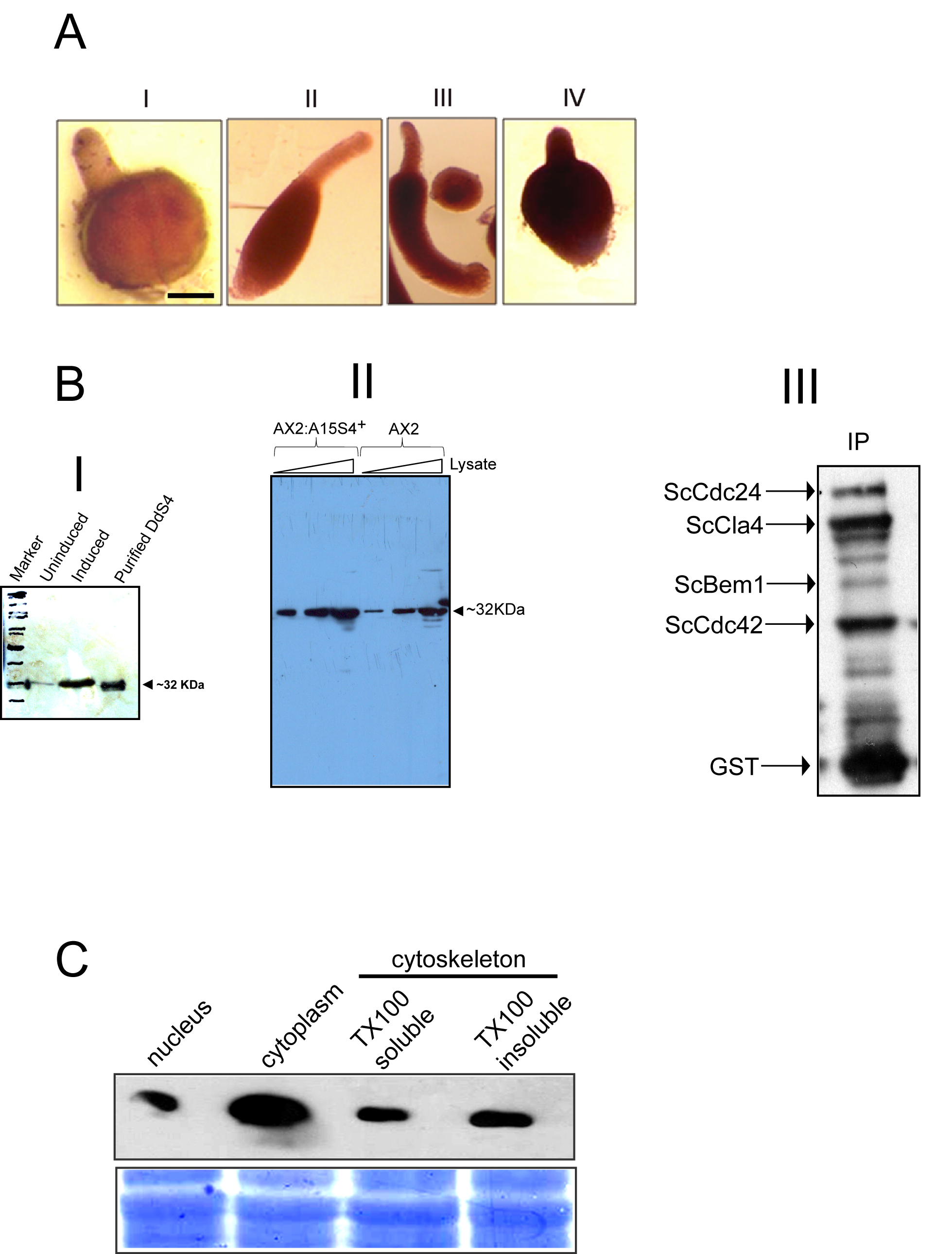

Supplement: Figure S2 — Spatial localization DdS4 mRNA, anti-DdS4 antibody specificity and sub-cellular localization of DdS4. (A) In situ hybridisation of developmental stages using a DdS4 DNA probe. (I) Tipped mound (II) Slug (III) Early culminant (IV) control using H3a DNA probe. (B) Specificity of anti-DdS4 antibody was determined by western blot using (I) E. coli BL21 lysate and probing with anti-His antibody (DdS4 is expressed as a His-tag protein) and (II) D. discoideum lysates where increasing amounts of lysates were loaded from either control AX2 or AX2::A15S4+. (III) Specific interactions between the GST-tagged proteins and DdS4 are observed in the IP lane. Refer Figure 2A legend for details. This is an over-exposed blot to visualize pull downs of Bem1p and Cla4p more clearly. (C) Subcellular fractionation of cells. Aggregating AX2 cells were subjected to high speed centrifugation to yield cytosolic and membrane fractions. The soluble and insoluble cytoskeletal fractions were also checked for DdS4. Equal loading was checked by Coomassie staining. (TIF) [file pone.0030644.s002.tif]

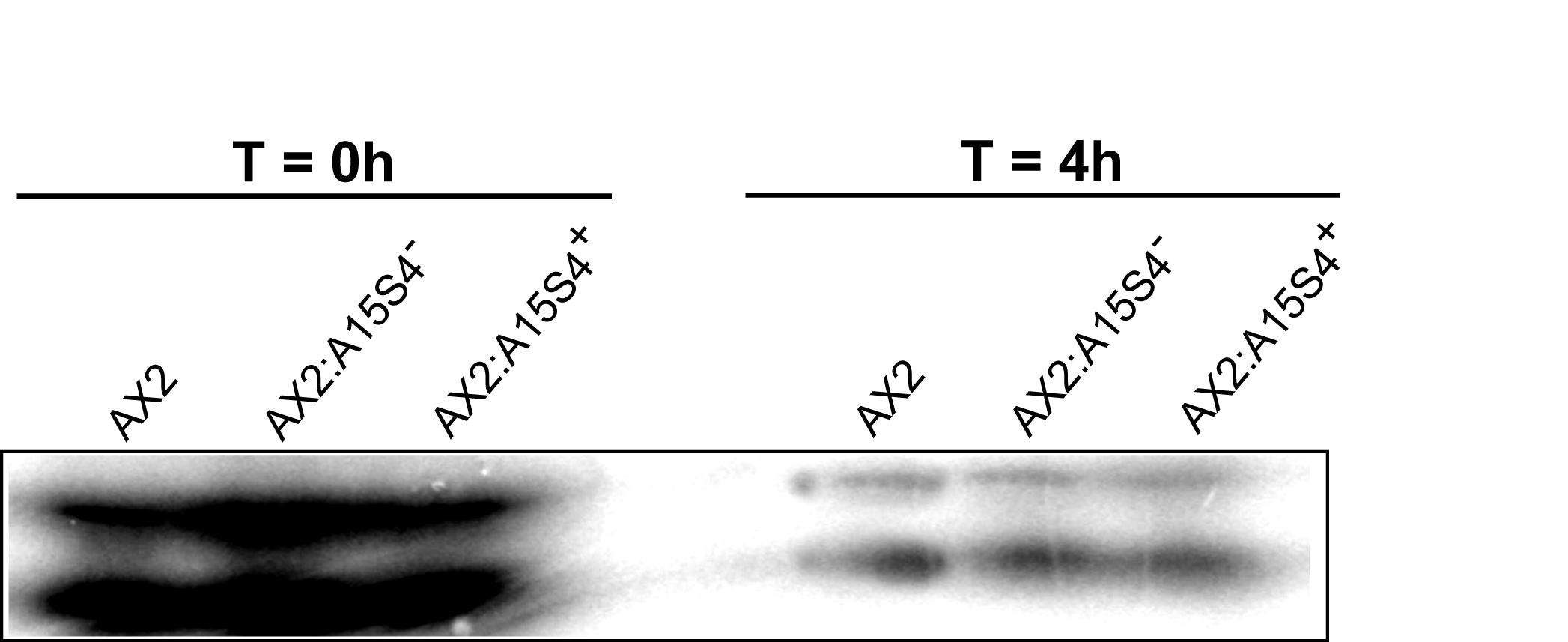

Supplement: Figure S3 — AX2:A15 mutants display normal protein synthesis. DdS4 cells were pulse-labelled for 1 h with [35S]methionine and chased for 4 h. The protein bands indicate equal rate of incorporation and subsequent chase of 35S label. (TIF) [file pone.0030644.s003.tif]

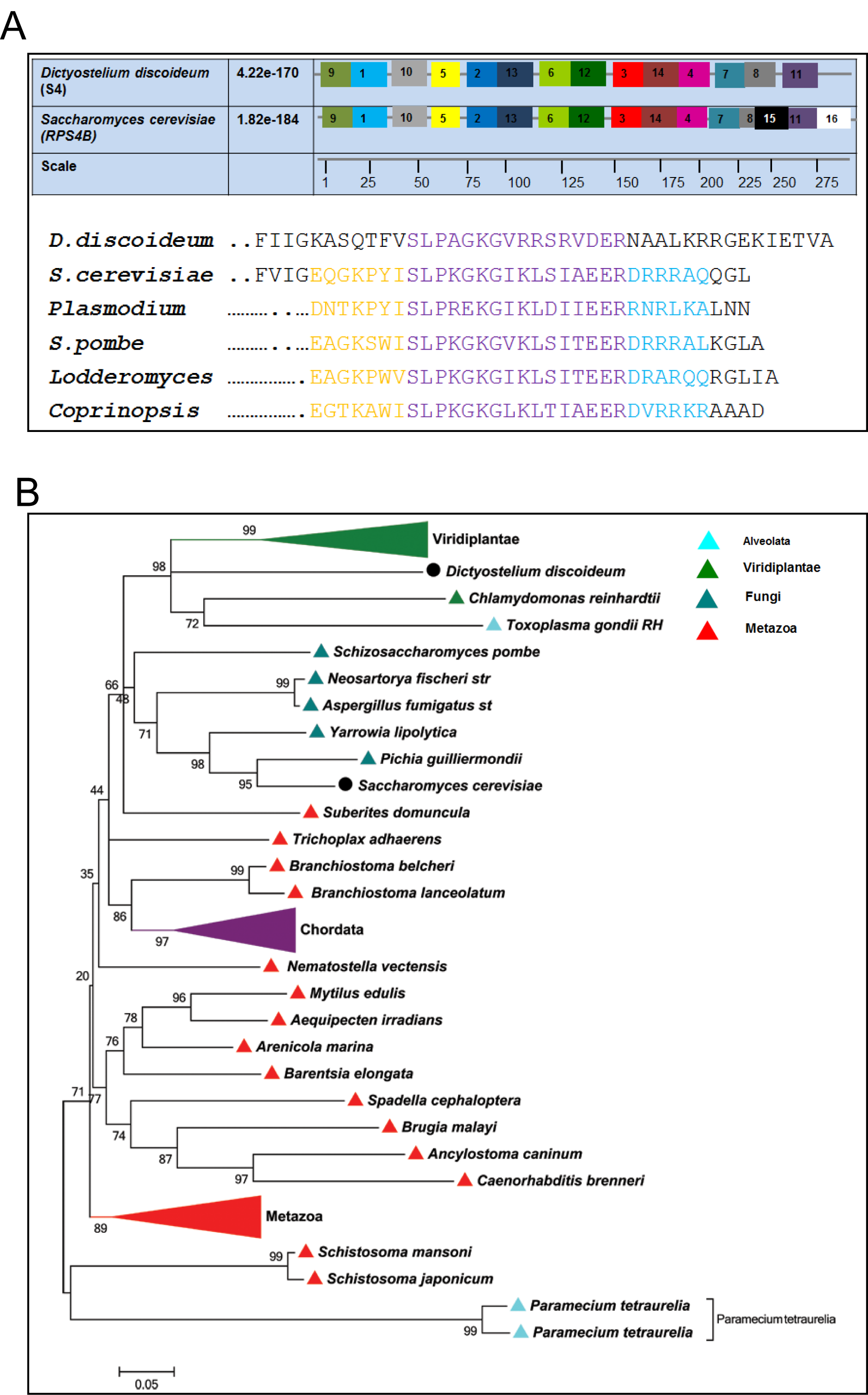

Supplement: Figure S4 — DdS4 is different from ScS4 at the C-terminal end. (A) Motifs conserved across S4 homologs (I). (II) The C-terminal region from few DdS4 homologs (S.cerevisiae, P.falciparum, S.pombe, L.elongisporus, C.cinerea okayama) depicting motif 11 (purple), 15 (yellow) and 16 (blue). S4 homolgs were submitted in MEME [80] to identify locally conserved regions (depicted by different colored rectangular boxes numbered according to the e-value, 1 showing the lowest e-value). N-terminus is largely conserved while variation largely lies in the C-terminus. DdS4 C-terminus lacks conserved motifs 15 and 16 ; purple region depicts motif 11 while yellow and blue regions show motifs 15 and 16 respectively. Motif 15 and 16 are not conserved in DdS4 showing a very high e-value. (B) Tree depicting all the S4 homologous sequences. All sequences considered are of eukaryotic origin. Tree was generated using Neighborhood joining method and validated by bootstrapping for 500 iterations (values indicated on the branches). Nodes have been clustered and color coded based on the kingdom, all the Metazoan sequences are represented in Purple, Plants in green and fungi in Blue. ScS4 (Dark Blue edge) clusters with other fungal S4 sequences (sky blue edge), DdS4 (dark blue edge) shows variation from other S4 (more similar to Plants). Tree generation and statistical testing done using MEGA [81] (TIF) [file pone.0030644.s004.tif]
